# Catalytic inactivation of influenza virus by iron oxide nanozyme

**DOI:** 10.7150/thno.35826

**Published:** 2019-09-21

**Authors:** Tao Qin, Ruonan Ma, Yinyan Yin, Xinyu Miao, Sujuan Chen, Kelong Fan, Juqun Xi, Qi Liu, Yunhao Gu, Yuncong Yin, Jiao Hu, Xiufan Liu, Daxin Peng, Lizeng Gao

**Affiliations:** 1 College of Veterinary Medicine, Yangzhou University, Yangzhou, Jiangsu, 225009, PR China.; 2 Institute of Translational Medicine, School of Medicine, Yangzhou University, Yangzhou, Jiangsu, 225001, PR China.; 3 Jiangsu Co-Innovation Center for the Prevention and Control of Important Animal Infectious Disease and Zoonoses, Yangzhou, Jiangsu, 225009, PR China.; 4 CAS Engineering Laboratory for Nanozyme, Institute of Biophysics, Chinese Academy of Sciences, Beijing, 100101, China.; 5 Jiangsu Research Centre of Engineering and Technology for Prevention and Control of Poultry Disease, Yangzhou, Jiangsu, 225009, PR China.; 6 Joint Laboratory Safety of International Cooperation of Agriculture&Agricultural-Products, Yangzhou, Jiangsu 225009, PR China.

**Keywords:** Iron oxide nanozyme, lipid peroxidation, lipoxidase-like activity, influenza virus, antivirus

## Abstract

Influenza poses a severe threat to human health in the world. However, developing a universal anti-viral strategy has remained challenging due to the presence of diverse subtypes as well as its high mutation rate, resulting in antigenic shift and drift. Here we developed an antiviral strategy using iron oxide nanozymes (IONzymes) to target the lipid envelope of the influenza virus.

**Methods:** We evaluated the antiviral activities of our IONzymes using a hemagglutination assay, together with a 50% tissue culture infectious doses (TCID_50_) method. Lipid peroxidation of the viral envelope was analyzed using a maleic dialdehyde (MDA) assay and transmission electron microscopy (TEM). The neighboring viral proteins were detected by western blotting.

**Results:** We show that IONzymes induce envelope lipid peroxidation and destroy the integrity of neighboring proteins, including hemagglutinin, neuraminidase, and matrix protein 1, causing the inactivation of influenza A viruses (IAVs). Furthermore, we show that our IONzymes possess a broad-spectrum antiviral activity on 12 subtypes of IAVs (H1~H12). Lastly, we demonstrate that applying IONzymes to a facemask improves the ability of virus protection against 3 important subtypes that pose a threat to human, including H1N1, H5N1, and H7N9 subtype.

**Conclusion:** Together, our results clearly demonstrate that IONzymes can catalyze lipid peroxidation of the viral lipid envelope to inactivate enveloped viruses and provide protection from viral transmission and infection.

## Introduction

Influenza A viruses (IAVs) are characterized by a particulate morphology with the size at 80-120 nm in diameter, consisting of segmented RNA, a typical glycoprotein-studded lipid envelope overlying a matrix protein 1 (M1). IAVs infect a broad range of species, including wild birds, poultry, and humans, resulting in annual flu epidemics and global pandemics^1^. Although various vaccines have been developed against IAVs, antigenic shift and drift accelerate the diversity and emergence of novel stains, rendering timely vaccine development a considerable challenge^2^. Antiviral drugs represent an alternative anti-AIVs strategy, however drug-resistance rapidly renders those drugs ineffective^3^. Moreover, the presence of multiple influenza subtypes renders developing a universal anti-IAVs strategy difficult. In contrast, all types of IAVs possess a lipid envelope as a conserved component essential for maintaining virus integrity and infectivity. Therefore, compromising the viral lipid envelope should result in inactivation of the virus, potentially representing an antiviral strategy against multiple subtypes of IAVs.

The development of nanotechnology has provided abundant nanomaterials for antiviral treatments^4^. Specifically, many inorganic nanoparticles have shown antiviral effects on influenza viruses, such as silver nanoparticles^5^ or gold nanoparticles^6^. However, how these nanomaterials affect viruses remains unclear. In recent years, the intrinsic enzyme-like properties of nanomaterials have raised widely concerns, providing in-depth understanding on the biological effects of nanomaterials. Now these nanomaterials performing enzyme-like activity are proposed as a new generation of artificial enzymes and denoted as “Nanozymes”^7^-^9^. The main property of nanozymes, compared to natural enzymes, is that their activity can be adjusted by modulating the size, morphology, dopant and surface modification 10, 11. Iron oxide (Fe_3_O_4_) nanoparticles, as the most typical nanozymes, which performs two enzyme-like activities, namely peroxidase and catalase activities^12^. This classical nanoparticle is currently used for bioseparation, biosensing, bioimaging, drug delivery as well as hyperthermia therapy, primarily because of its unique magnetic properties 12. Previously, we found that at acidic pH conditions, ferromagnetic nanoparticles exhibit peroxidase-like activities, which can catalyze the reaction of hydrogen peroxide into hydroxyl radicals^13^. Due to this unique enzyme-like property, iron oxide nanoparticles are denoted as iron oxide nanozymes (IONzymes). We consequently applied this catalytic property to biomarker detection in immunoassay, H_2_O_2_ and Glucose Detection^14^, Ebola detection^15^, tumor diagnosis^16^, anti-bacteria and biofilm elimination for caries prevention^17^, ^18^ and wound healing^19^. In addition, IONzymes were shown to exhibit catalase-like activity at neutral pH conditions, converting hydrogen peroxide into oxygen^20^. These catalase-like activities enable IONzymes to reduce reactive oxygen species (ROS), resulting in protection of neurons and improving anti-inflammatory processes^21^. A recent study also reported that IONzymes induce membrane lipid peroxidation in synthesized liposome^22^, ^23^. On the basis of these earlier observations, we hypothesized that IONzymes may provide a possibility to develop universal antiviral strategy and represent a suitable tool for investigating the antiviral mechanisms of inorganic nanomaterials.

**Scheme 1:**
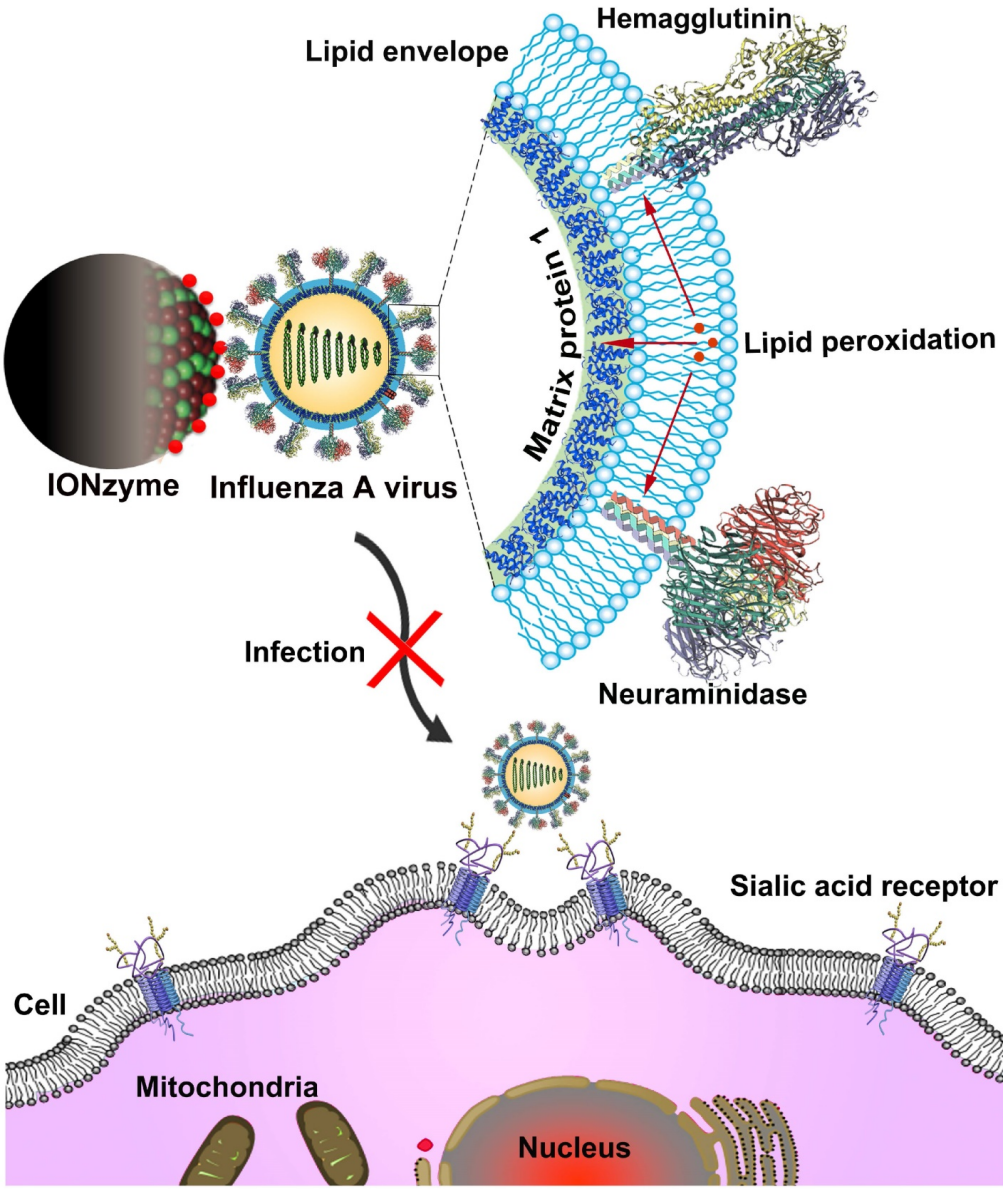
Schematic of viral liperoxidation by IONzymes for virus inactivation. IONzymes directly contact with IAVs particles and collapses viral lipid envelope via enhancing the level of lipid peroxidation, which further produces free radicals to destroy the neighboring proteins, including hemagglutinin, neuraminidase, and matrix protein 1, and then impaired various viral structures and functions, resulting in a failed infection into host cells.

Here, we present a successful paradigm of broad-spectrum antiviral activity on IAVs using IONzymes. We found that our IONzymes catalyze lipid peroxidation of the viral lipid envelope and thus compromise the integrity of the neighboring proteins (Scheme 1). In addition, the catalytic inactivation mediated by IONzymes act on variable subtypes of IAVs, showing the ability to prevent IAVs transmission and infection.

## Results

### IONzymes induce lipid peroxidation to destruct Influenza A viruses

We synthesized IONzymes using a solvothermal method, a common system to produce inorganic nanocrystal products. As shown in Figures S1, the synthesized IONzymes measured approx. 200 nm in diameter. Importantly, it exhibited a high peroxidase-like activity as demonstrated by the colorimetric reaction of hydrogen peroxide (H_2_O_2_) (Figure S2) and 3,3',5,5'-tetramethylbenzidine (TMB) (Figure S3) under acidic condition. The catalytic kinetics followed typical Michaelis-Menten kinetics. The Vmax values to H_2_O_2_ and TMB were 207 nM/s and 263.5 nM/s, respectively (Table S1). These features demonstrated that the quality of IONzyme products are identical to those prepared before^13^.

**Figure 1:**
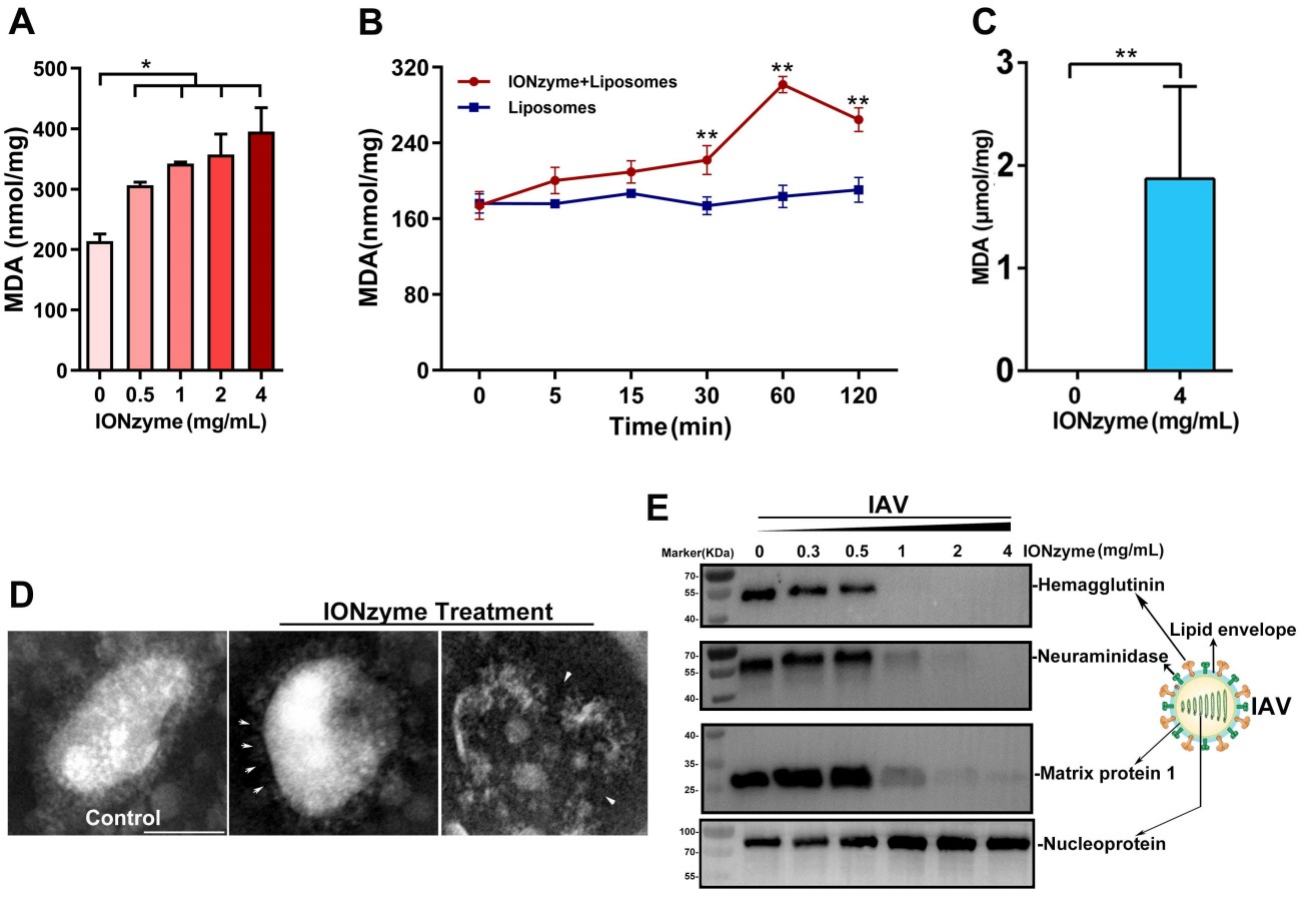
** IONzymes compromise the lipid envelope and neighboring proteins of IAVs through lipid peroxidation. (A-B)** The level of lipid peroxidation (MDA detection) when liposomes were treated by IONzymes, **(C)** or when IAVs (H5N1 SY strain) treated by IONzymes. **(D)** TEM image of IAVs treated by IONzymes (4 mg/mL). The left image: untreated IAVs. The asterisk: the destroyed lipid envelope of virion. Scale bar: 50 μm. **(E)** Western blot analysis of hemagglutinin, neuraminidase, matrix protein 1, and nucleoprotein protein of IAVs treated by IONzymes. All experiments were repeated in triplicate with a representative image shown. Results are as means±s.d. Statistical significance is assessed by unpaired Student's two-sided *t*-test to the control group. *P<0.05. **P<0.01.

To investigate whether IONzymes are capable to induce lipid peroxidation, we measured maleic dialdehyde (MDA) produced by incubating our IONzymes with liposome constituted with egg phospholipid. As shown in Figure 1A, IONzymes at 4 mg/mL with 2 h incubation resulted in increased MDA levels up to 2 folds compared to untreated liposome. In addition, the generation of MDA presented a dependence on IONzymes dosage at neutral pH conditions (Figure 1A) and reached a peak value at 1 h (Figure 1B).

Together, these results indicated that IONzymes have the ability to induce lipid peroxidation in liposomes. Importantly, lipid peroxidation occurred under neutral condition rather than acidic pH, indicating that catalytic performance resembles lipoxidase activity rather than peroxidase, which requires acidic pH and the presence of hydrogen peroxide.

Next, we tested whether IONzymes induce lipid peroxidation on the viral envelope of influenza A virus that possesses a liposome-like structure (named henceforth viral lipid envelope). To this end, we incubated IAVs (H5N1 subtype, SY strain, if not specified) with IONzymes at physiological conditions. As shown in Figure 1C and S4, incubation with IONzymes at 4 mg/mL for 2 h resulted in 1.87 ± 1.16 µmol/mg MDA, demonstrating that IONzymes are able to induce lipid peroxidation of the viral envelope of IAVs at neutral pH. Liposome is simply constituted with lipid. The commercial lipid may have been oxidized in the storage and thus already has certain level of MDA before forming liposome. In contrast, a virus is composed of lipid, proteins and nucleic acids, forming a rigid structure with proteins on viral surface. Therefore, no signal was detected in IONzyme-untreated IAVs, presumably because that viruses rather than liposomes possess considerable structural stability under natural conditions.

In agreement with these results, examining viral morphology by transmission electron microscope (TEM) showed that the lipid envelope of IONzyme-pretreated IAVs was considerably damaged (Figure 1D). In addition, we examined the neighboring proteins of IAVs, including hemagglutinin, neuraminidase (NA), matrix protein 1 (M1), and nucleoprotein (NP). As shown in Figure 1E, hemagglutinin, NA, and M1 proteins were destructed under high concentration of IONzymes (> 1 mg/mL), whereas NP protein appeared to be unaffected. It is well known that hemagglutinin and NA have transmembrane domains in viral lipid and M1 is close to inside of the lipid. It is reasonable that these protein destructions were ascribed to lipid peroxidation. To confirm the relationship between protein destruction and lipid peroxidation, we investigated the extent of the damage of bovine serum albumin (BSA), as a standard protein, in the IONzyme-liposome mixture under neutral pH. As shown in SDS-PAGE result (Figure. S5), BSA was well degraded by IONzyme-liposome system other than single IONzymes. We then tested purified hemagglutinin protein using the same IONzyme-liposome system. The subsequent western blot assay showed that hemagglutinin protein was destructed (Figure S6). Together, these findings indicated that lipid peroxidation in liposome induced by IONzymes was able to degrade proteins. Therefore, we concluded that IONzymes induce lipid peroxidation of the viral lipid envelope, and simultaneously compromise the integrity of envelope structure as well as the integrity of neighboring proteins of H5N1 virus.

### IONzymes destruct hemagglutination activity of IAVs

**Figure 2:**
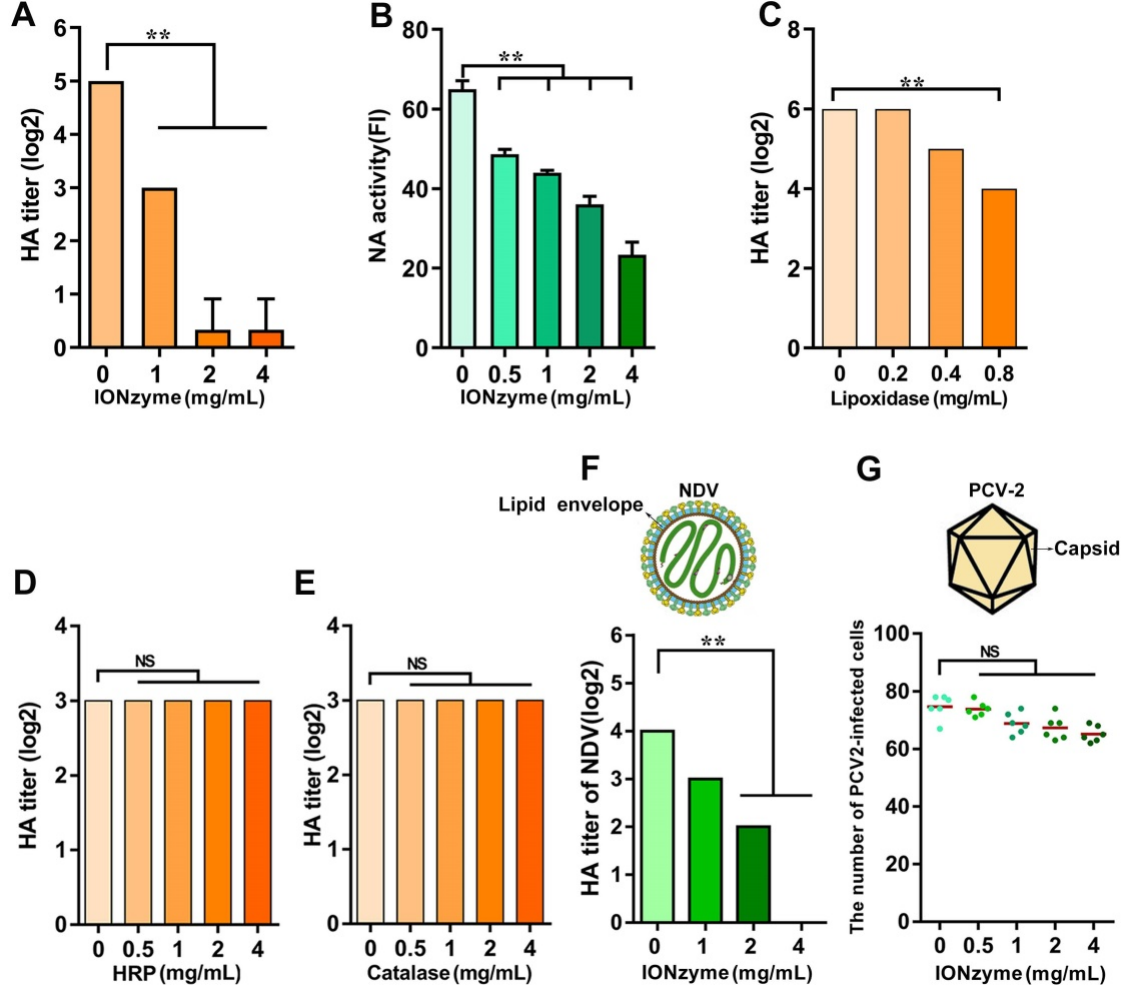
** IONzymes destruct hemagglutination activity of IAVs. (A)** HA titer of IONzyme-treated IAVs (H5N1 SY strain). **(B)** NA activity of IONzyme-treated IAVs. FI, fluorescence intensity. **(C, D, E)** HA titer of lipoxidase (C, Sigma, L7395), or peroxidase (D, HRP, Solarbio, P8020), or catalase (E, Beyotime, S0082)-treated IAVs, under 2 h, 37 ℃, neutral pH. **(F, G)** Antiviral activity of IONzymes against envelope virus and non-enveloped virus. Different concentrations of IONzymes were mixed with NDV (F, representative envelope virus) and PCV-2 (G, representative non-enveloped virus) for 2 h. The magnet was placed under the pipes to pull down IONzymes and then supernatant was collected to detect HA titer of NDV and the number of PCV2-infected PK15 cells by immunofluorescence assay. Each dot represents the total number of infected cells obtained from one image (n=6). Horizontal lines across the scatter plot represent mean values. All experiments were repeated in triplicate with a representative image shown. Results are as means±s.d. Statistical significance is assessed by unpaired Student's two-sided *t*-test to the control group. **P<0.01. *NS* represents no significant difference.

To evaluate the activity of hemagglutinin on H5N1 virus after treatment with IONzymes, we performed hemagglutination (HA) assay, which is indexed in HA titer to reflect the function of hemagglutinin protein^24^. We found that IONzymes reduce the HA titer of either chicken embryos-produced (Figure 2A, S7) as well as cell-produced H5N1 virus (Figure S8) as well as further purified virus (Figure S9), showing that the reduction occurs in a dose and time-dependent manner (Figure S9). In addition, IONzymes with 200 nm at diameter posed a stronger lipid peroxidation level (Figure S10) and antiviral activity (Figure S11) than those with diameter at 100 nm or 50 nm. As shown in Figure 2A, the HA titer (Log2) of H5N1 virus was reduced from 5.00±0.00 to 0.33±0.57 when IAVs were treated with 4 mg/mL of IONzymes (200 nm at diameter, unless specified otherwise) under 2 h. These findings indicated that hemagglutinin protein lost the ability to bind sialic acid receptors on blood cells in IONzyme-treated H5N1 virus. NA function also influences hemagglutination activity because of a functional balance between the hemagglutinin and NA protein^25^. Similarly, more than 60% of NA activity was reduced when H5N1 virus was treated with 4 mg/mL of IONzymes under 2 h (Figure 2B). These results indicated that the biological functions of hemagglutinin and NA protein were compromised by IONzymes.

To confirm that such inactivation was caused by lipoxidase catalysis, we compared the effects of natural lipoxidase treatment on H5N1 virus using the same procedure in IONzyme experiments. As shown in Figure 2C, natural lipoxidase at 0.8 mg/mL reduced HA titer (Log2) from 6 to 3.8, indicating that natural lipoxidase induced denaturation of H5N1 virus in a manner similar to IONzymes. Furthermore, IONzymes possessed a well antiviral activity under O_2_-rich conditions (Figure S12). However, both natural peroxidase (horseradish peroxidase, HRP) and catalase failed to show such reduction in HA titer (Log2) (Figure 2D, E). These results indicated that IONzymes destruct viruses probably through mimicking the lipoxidase-like activity rather than peroxidase-like or catalase-like activity.

To confirm the contribution of lipid peroxidation in viral inactivation, we compared the effects of IONzymes between enveloped viruses and non-enveloped viruses. Two viruses, Newcastle disease virus (NDV, representative envelope virus) and Porcine circovirus type 2 (PCV-2, representative non-enveloped virus) were treated with IONzymes following the same procedure for H5N1 virus. As shown in Figure 2F, IONzymes treatment (4 mg/mL) reduced the HA titer (log 2) of NDV below levels of detection, with similar effects found for H5N1 virus. In contrast, the same IONzymes treatment only reduced 16% infectivity (number of infected cells) of PCV-2 (Figure 2G, Figure S13). Together, these results demonstrated that the lipid envelope is the target of IONzymes.

**Figure 3:**
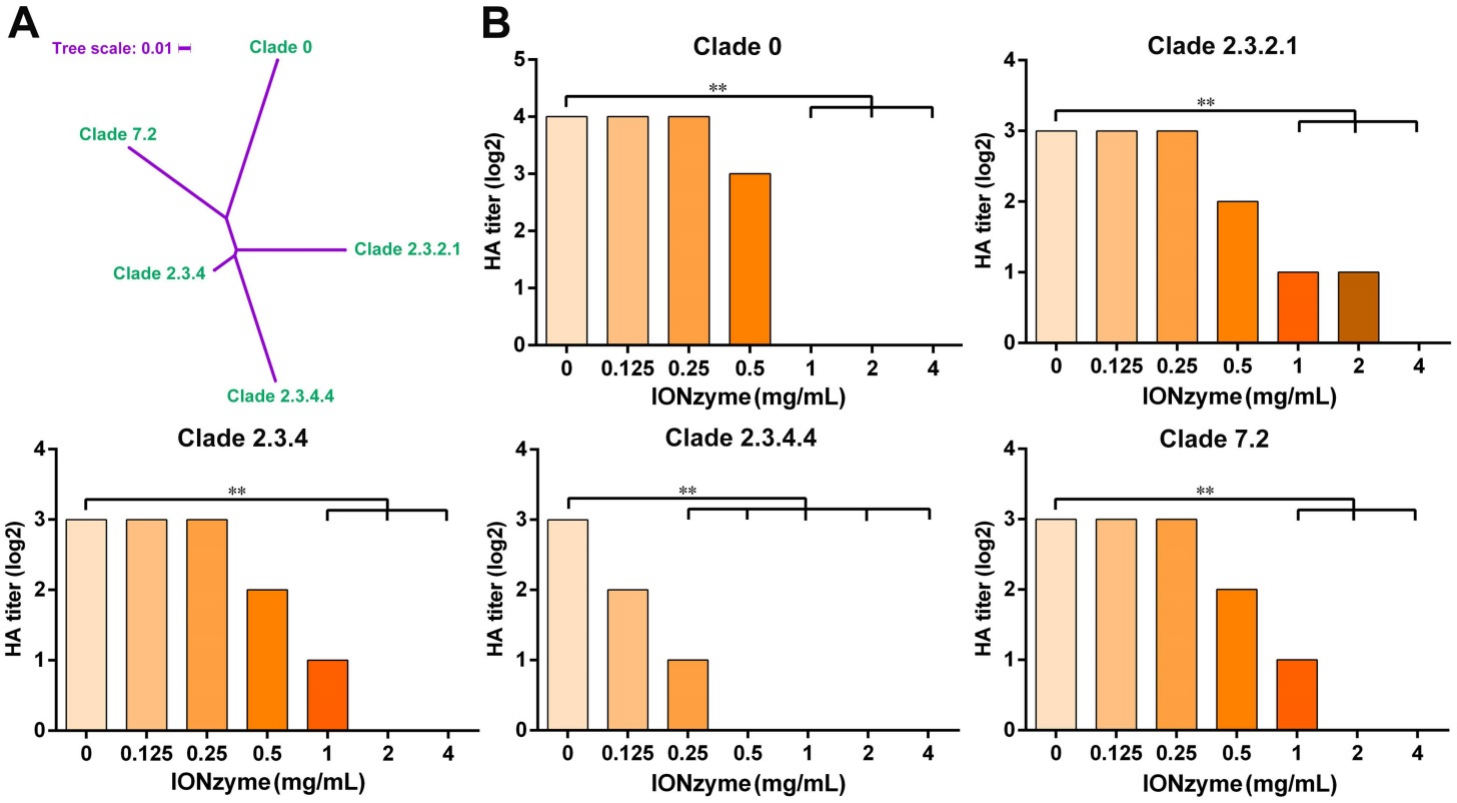
** IONzymes destruct hemagglutination activity of multiple H5 clades. (A)** Phylogenetic analysis of the complete hemagglutinin genes of H5 subtype IAVs from different clades, including clade 0, clade 2.3.2.1, clade 2.3.4, clade 2.3.4.4, and clade 7.2. The complete sequences were chosen to perform multiple sequence alignment by MEGA (Version 6), and then, maximum likelihood phylogenetic trees were inferred with 1,000 bootstraps. The result is displayed as a dendrogram using iTOL (Interactive Tree Of Life). **(B)** HA titer of IONzyme-treated H5 clades, under 2 h. Data shown represent the means±s.d. of three independent experiments. Statistical significance is assessed by unpaired Student's two-sided *t*-test to the control group (IONzymes, 0 mg/mL). **P<0.01.

H5 subtype IAV mutates much more rapidly than most other subtypes such as H7 and H9. Therefore, a standardized “clade” nomenclature was developed and first adopted in 2008, based on the evolution and divergence of H5N1 viruses that evolved from the original HA gene of the 1996 H5N1 virus. To date, 10 distinct clades (0-9) and many sub-clades have been identified^26^. Here, we further checked the envelope-dependent virus destruction using H5 clades (Figure 3A). In addition to the H5N1 SY strain, we tested the effect of IONzymes on other representative H5 strains originated from different clades, including clade 0, clade 2.3.2.1, clade 2.3.4, clade 2.3.4.4, and clade 7.2, which possess the same envelope structure except genetic mutations of H5 hemagglutinin protein. As shown in Figure 3B, IONzymes treatment dramatically reduced HA titer (Log2) for all H5N1 clades. In particular, HA titer (Log2) was all undetectable in the group treated with 4 mg/mL IONzymes under 2 h. These results demonstrated that IONzymes treatment can destruct a virus with a common envelope structure such as H5N1, despite the fact that it contains numerous clades.

### IONzymes abolish infectivity of IAVs

**Figure 4:**
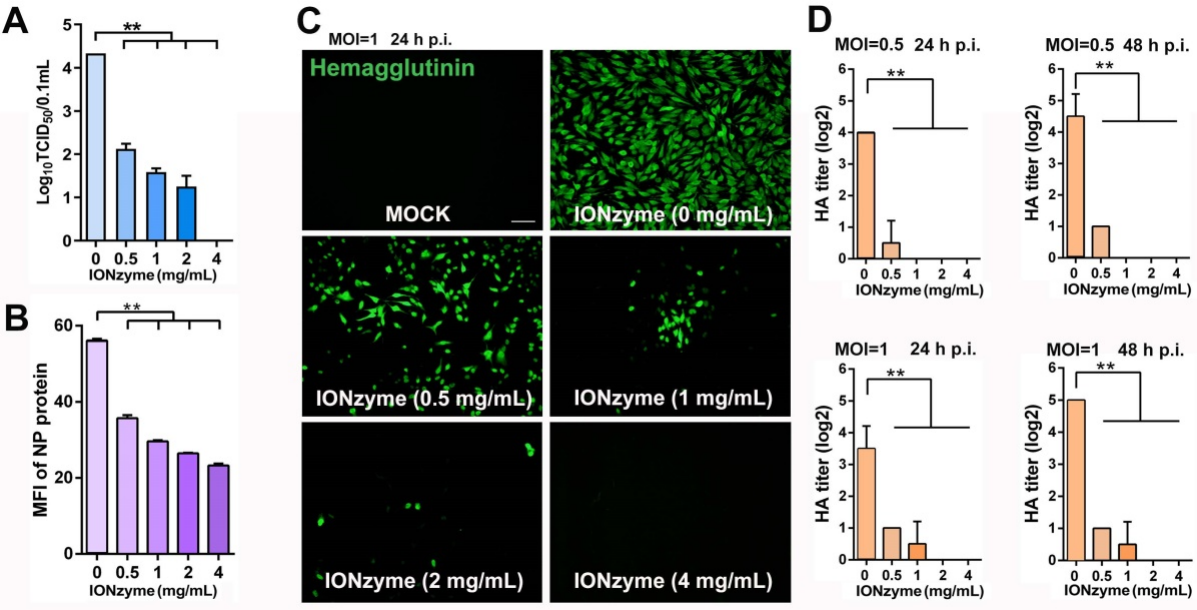
** IONzymes abolish infectivity of IAVs. (A)** TCID_50_ titer of IONzyme-treated IAVs (H5N1 SY strain) under 2 h. **(B)** Attachment ability of IONzyme-treated IAVs (pretreated for 2 h) on MDCK cells under 1 h. NP protein was detected by flow cytometry. MFI, mean fluorescence intensity. **(C, D)** MDCK cells were infected with IONzyme-treated IAVs at a MOI of 0.5 or 1. **(C)** At 24 h p.i., the cells were fixed to detect replication by immunofluorescence stain of hemagglutinin protein (green).** (D)** Supernatants were collected at 24 and 48 h p.i. to detect viral release by HA titer. Data shown represent the means±s.d. of three independent experiments. Statistical significance is assessed by unpaired Student's two-sided* t*-test to the control group. *P<0.05; **P<0.01. Scale bar: 50 μm.

Next, we evaluated the infectivity of IAVs treated with IONzymes. To exclude possible interference from other components present in either the allantoic fluid or cell lysate, we purified H5N1 virus using a discontinuous sucrose density gradient. The purified H5N1 virus was incubated with IONzymes and then collected for the assay of 50% tissue culture infectious doses (TCID_50_) in Madin-Darby canine kidney (MDCK) cells^27^, ^28^. As shown in Figure 4A, the value of Log_10_ TCID_50_ per 0.1 mL was reduced from 4.33±0.00 (without treatment) to below the detection limit (with 4 mg/mL of IONzymes under 2 h), indicating that H5N1 virus treated by IONzymes lost its infectivity.

We next examined the attachment, proliferation, and release of H5N1 virus in the process of infection to MDCK cells. We quantified virus attachment on the surface of MDCK cells by using NP protein as marker, as it was the only protein not affected upon IONzymes treatment. To measure NP protein, virus samples were incubated with MDCK cells for 1 h and then labeled with fluorescent anti-NP antibody for flow cytometer assay. As shown in Figure 4B and Figure S14, the value of mean fluorescence intensity (MFI) of NP protein was 23.2±0.91 in the virus treated with 4 mg/mL IONzymes, which was much lower than the value of 56.1±0.73 in the intact virus. This result demonstrated that the ability of H5N1 virus attach to MDCK cells was reduced, which can be ascribed to the destruction of hemagglutinin by IONzymes. We next examined the sequential viral replication of IONzyme-treated H5N1 virus in MDCK cells. As shown in Figure 4C, the intact H5N1 virus efficiently replicated inside MDCK cells (labelled with anti-hemagglutinin antibody) at 24 h post infection (p.i.) when the value of multiplicity of infection (MOI) was set to 1 (the ratio of infectious virus to target cells). In comparison, no fluorescent signal was observed in the virus treated with IONzymes at 4 mg/mL for 2 h, indicating that viruses failed to replicate in MDCK cells. This result may be due to failed invasion of H5N1 virus following treatment with IONzymes. We next evaluated the viral titer (HA titer) of budding viruses released into the supernatant of cell culture when H5N1 virus infected MDCK cells. As shown in Figure 4D, the HA titer (Log2) became undetectable when virus was treated with 2 mg/mL IONzymes for 24 or 48 h p.i.. This result indicated that IONzymes treatment may stop the production of progeny virions due to the reduction of NA activity. Taken together, IONzymes abolish the infectivity of H5N1 virus to host cells, especially at the initial attachment and invasion step of viral proliferation process^29^.

### IONzymes reduce virulence and pathogenicity of IAVs

**Figure 5:**
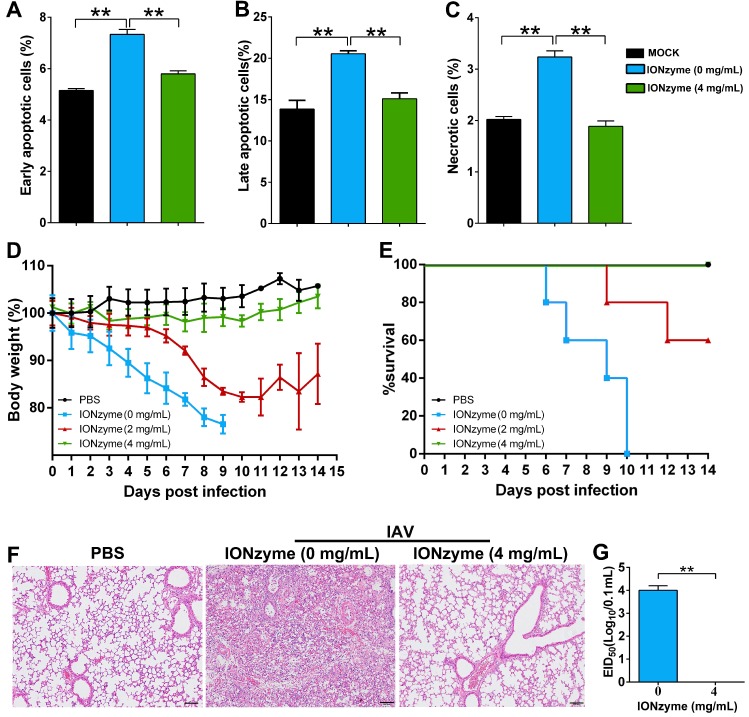
** IONzymes reduce IAVs-induced apoptosis *in vitro* and pathogenicity *in vivo*. (A-C)** Apoptosis analysis of MDCK cells infected with IONzyme-treated IAVs (H5N1 SY strain) using flow cytometry. MOI = 1 under 24 h p.i.. The proportions of early apoptotic cells (annexin V+ and PI-; A), later apoptotic cells (annexin V+ and PI+; B), and necrotic cells (annexin V- and PI+; C) were analyzed. **(D-G)** BALB/c mice were challenged intranasally with IONzyme-treated of IAVs. **(D)** Morbidity was evaluated by monitoring weight changes over a 14-day period and was plotted as a percentage of the animals' weights on the day of inoculation (day 0). The data represent the average body weight of each group (n = 5). **(E)** Mortality expressed as percent survival. **(F)** Representative histopathological changes in H&E (hematoxylin and eosin)-stained lung tissues on day 7 p.i.. **(G)** Viral replication in the lung (n = 3). Data shown represent the means±s.d. of three samples. Statistical significance is assessed by unpaired Student's two-sided t-test (two groups). **P<0.01. Scale bars: 500 μm.

We next evaluated the virulence and pathogenicity of H5N1 virus treated with IONzymes. Influenza virus infection induces apoptosis in multiple cell types, subsequently resulting in cellular and organ damage, leading to lethal pathogenicity 30. To determine cell damage, we inoculated MDCK cells with H5N1 virus with or without prior treatment with IONzymes. We identified cellular apoptosis by double staining with annexin V and propidium iodide (PI) for flow cytometer assay. As shown in Figure 5A-C and S15, the infection of H5N1 virus primarily induced cell apoptosis rather than necrosis. H5N1 virus led to 20.6± 0.4 % cells with late apoptosis. In contrast, IONzyme (4 mg/mL)-treated H5N1 virus induced 15.1± 0.7 % cells with late apoptosis which is close to the same level in mock cells (without virus infection), indicating IONzymes treatment reduced the virulence of H5N1 virus* in vitro.*

We further evaluated the pathogenicity of IONzyme-treated H5N1 virus using an animal-infection model. To this end, we chose mice (BALB/c) due to their high susceptibility to H5N1 virus as well as its high pathogenicity^31^. The infection of intact H5N1 virus reduced the body weight of mice by over 20%, whereas those infected with H5N1 virus treated with 4 mg/mL IONzymes maintained their body weight, similar to uninfected mice (Figure 5D). We also observed highly mortality in the mice infected with intact H5N1 virus, in which all mice died within 10 days post infection (Figure 5E). In comparison, the mice infected with H5N1 virus treated with 4 mg/mL IONzymes obtained 100% survival rate in 14 days observation, identical to that in uninfected mice. We also examined the pathological status and viral proliferation in the lung tissue of the infected mice. Using an H&E staining method, we found that the lung tissues of mice infected with intact H5N1 virus exhibited severe pneumonia with inflammatory cellular infiltration, alveolar wall edema and thickening, and hemorrhaging on day 7 p. i. (Figure 5F). In comparison, the group infected with IONzyme (4 mg/mL)-treated H5N1 virus showed negligible pathological changes. We then conducted a 50% embryo infectious doses (EID_50_) assay in specific-pathogen-free (SPF) chicken embryo infection model. As shown in Figure 5G, no virus was detected in the lung tissue indicated by EID_50_ assay in the group infected with IONzyme-treated H5N1 virus. In comparison, mice infected with intact H5N1 virus showed the EID_50_ (Log_10_/0.1 mL) at 4.0±0.2 in the lung tissue. Taken together, these results showed that IONzymes efficiently inhibit IAVs-induced apoptosis in MDCK cells and reduce the pathogenicity and viral proliferation in mice.

### IONzymes destruct multiple subtypes of IAVs

**Figure 6:**
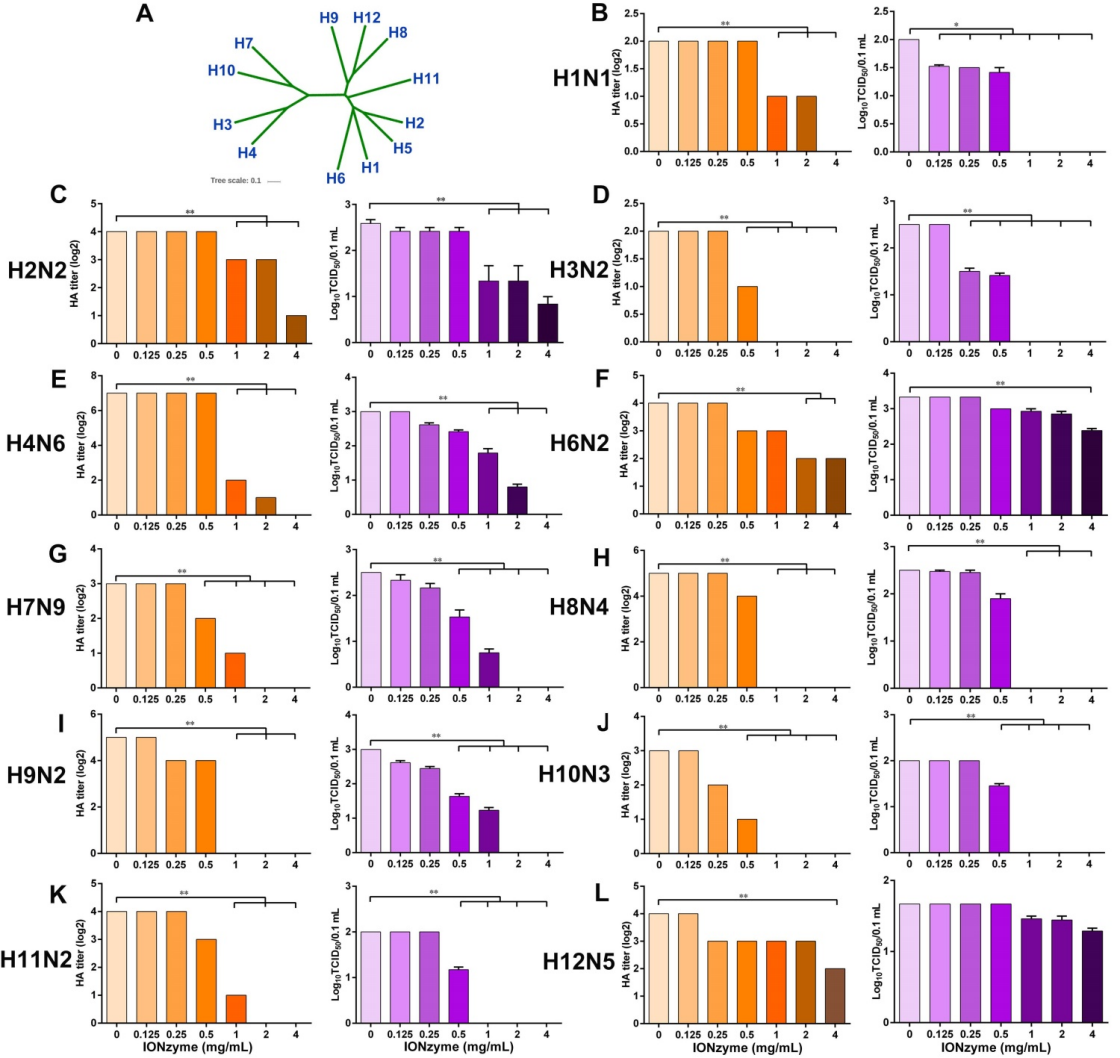
** Destruction of multiple subtypes of IAVs by IONzymes. (A)** Phylogenetic analysis of the complete hemagglutinin genes of H1~H12 subtype IAVs. The complete sequences were chosen to perform multiple sequence alignment by MEGA (Version 6), and maximum likelihood phylogenetic trees were inferred with 1,000 bootstraps. The results are displayed as a dendrogram using iTOL (Interactive Tree of Life). **(B-L)** HA and TCID50 titers of IONzyme-treated multiple subtype IAVs under 2 h, including H1(B), H2(C), H3(D), H4(E), H6(F), H7(G), H8(H), H9(I), H10(J) H11(K), and H12(L). Data shown represent the means±s.d. of three independent experiments. Statistical significance is assessed by unpaired Student's two-sided t-test to the control group. *P<0.05; **P<0.01.

To investigate the destructive effects on other subtypes of IAVs, we treated IONzymes with H1, H2, H3, H4, H6, H7, H8, H9, H10, H11, and H12 subtype and evaluated the HA and TCID_50_ titers. Although these subtypes are genetically distant according to phylogenetic analysis of the complete hemagglutinin genes (Figure 6A), IONzymes efficiently reduced the HA and TCID_50_ titers of above H1-H12 subtypes with dose-dependence (Figure 6B-L).

When treated with 2 mg/mL IONzymes, H7N9, a subtype of avian influenza which was first found in 2013 in China and causes severe illness, combined with high mortality^32^, ^33^, showed undetectable HA titer (Log2) and TCID_50_ (Log_10_/0.1 mL) (Figure 6G). Other subtypes, such as H3N2, H8N4, H9N2, H10N3, and H11N2, exhibited a similar destructive efficiency by IONzymes ( ≥2 mg/mL). If increased IONzymes up to 4 mg/mL for the treatment, H1N1, another threatening subtype which was from swine flu but infected many people in the world in 2009^34^, was completely inactivated according to the data of HA titer and TCID_50_ (Figure 6B). In fact, IONzymes at 4 mg/mL abolished biological activity of most subtypes except H2N2, H6N2, and H12N5. Collectively, the above results demonstrated that IONzymes possess a broad-spectrum effect on various subtypes of IAVs.

### IONzyme-loaded facemask protects against influenza

**Figure 7:**
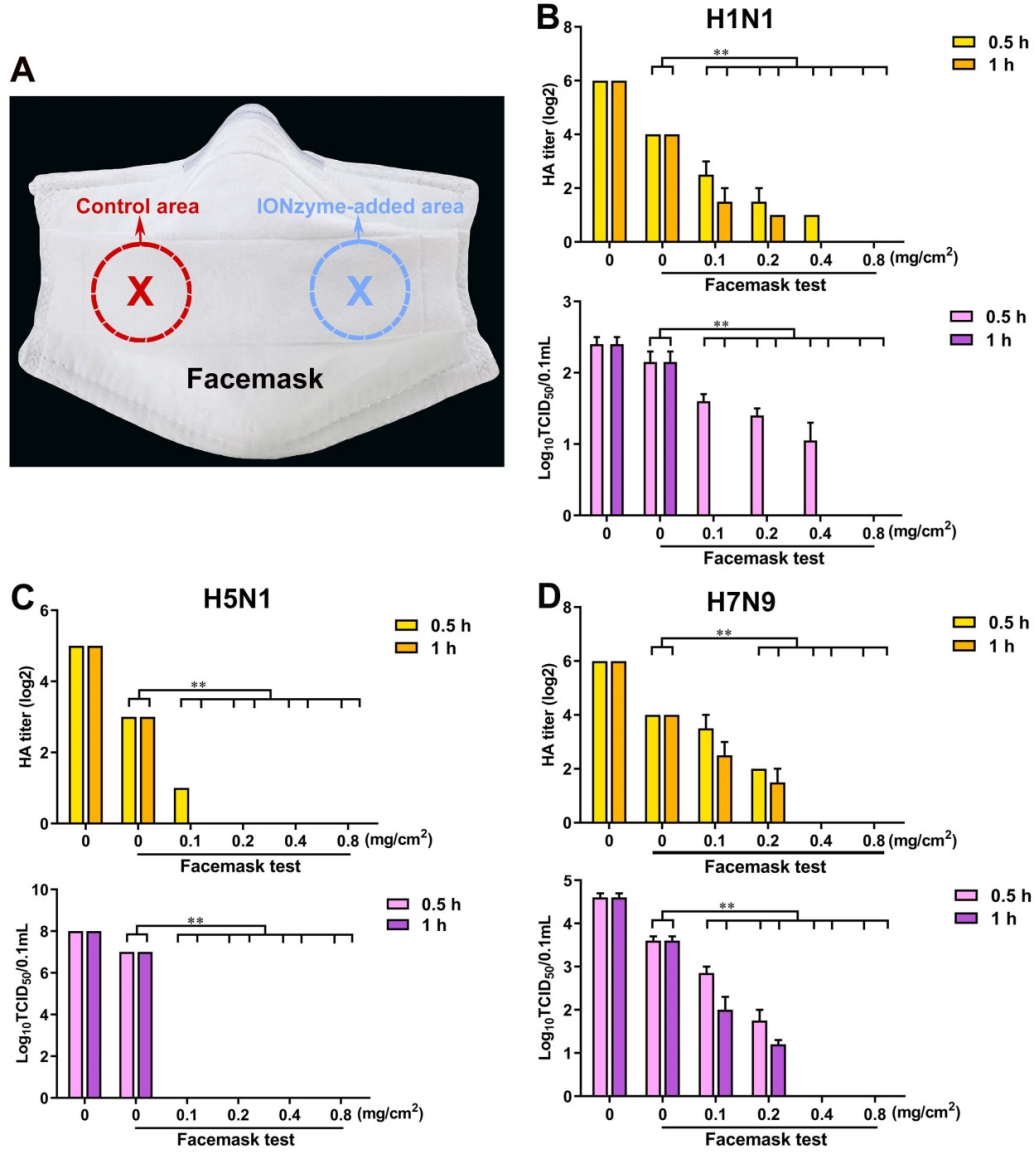
** IONzyme-loaded facemask protects against influenza. (A)** A picture of anti-IAVs mask with eight-layer absorbent filter cotton. Different concentrations of IONzymes were added on the third layer of selected area (5 cm^2^), respectively. After drying, IAVs (H1N1, H5N1, and H7N9 subtype) was sprayed onto the eighth layer (the outermost layer). After standing for 0.5 or 1 h, the population of the living virus on the control area or IONzyme-added area were harvested, and then HA and TCID_50_ titers of H1N1** (B)**, H5N1** (C)**, and H7N9** (D)** IAVs were measured. The data are presented as the mean ± s.d. of three independent experiments. Statistical significance is assessed by unpaired Student's two-sided* t*-test to the control group. **P<0.01.

Given the fact that IONzymes effectively abolish virus activity, we hypothesized that it might represent a suitable candidate to prevent the spread of active influenza virus, especially for representative IAVs that have a serious threat to human, including H1N1, H5N1, and H7N9 subtype. To test this hypothesis, we added IONzymes as an antiviral component into facemasks (Figure 7A). As shown in Figure 7B, in the control area, the HA titers (Log2) were 4.0±0.0 when allowing H1N1 virus sample (the titer at 6.0±0.0) to spray on the facemask for 0.5 or 1 h. Using a TCID50 assay, we measured the value of Log10 TCID50/0.1 mL as 2.2±0.2, which was comparable to the original H1N1 virus sample whose Log10 TCID50/0.1 mL was 2.4±0.1. These data and the results from H5N1 and H7N9 virus (Figure 7C, D) demonstrated that IONzyme-unloaded facemasks only offer a limit virus interception. In comparison, the IONzyme-added area of facemask showed effective inactivation of H5N1 virus. When IONzymes were added at 0.1 mg/cm2, both HA titers and TCID50 titers became undetectable after 1 h. If IONzymes were added above 0.2 mg/cm2, we detected no viral titers after 0.5 h (Figure 7C). If increased IONzymes up to 0.4 mg/cm2, both H1N1 and H7N9 virus were completely inactivated after 1 h (Figure 7B, D). Together, our results show that IONzymes greatly improve the protective function of facemasks, namely by abolishing viral activity. Importantly, storing masks for up to four weeks failed to diminish antiviral efficacy (Figure S16). Therefore, IONzymes represent an ideal antiviral additive that improves the protective efficiency against IAVs in personal protective equipments (PPEs) such as facemasks, bioprotective suits, as well as air/water filters applied in high-risk areas.

## Discussion

Our work provided a promising antiviral strategy using IONzymes to inactivate IAVs. As a potential antiviral alternative, IONzymes demonstrate the following features: (i) IONzymes inactivate IAVs by catalyzing viral lipid envelope and neighboring structure proteins through lipid peroxidation. (ii) IONzymes possess a broad spectrum anti-IAVs activity. (iii) IONzymes can be integrated into facemask to improve protection efficacy. These features together provide potential advantages for practical applications of IONzymes in biomedicine and consumer healthcare.

This finding extended biomedical applications of iron oxide nanoparticles with enzyme catalytic property. The discovery of intrinsic enzyme-like activities enables iron oxide nanoparticles to be used as enzyme mimics in biological system, including stimulating stem cell proliferation, killing bacteria and antioxidant protection^17^, ^35^, ^36^. In particular, although sporadic work observed that iron oxide nanoparticles showed certain antiviral effect on H1N1^37^, it remains far from clear how and why iron oxide nanoparticles destruct virus. In our antiviral experiment, we found that natural lipoxidase (non-heme iron-containing enzyme) rather than peroxidase or catalase performed antiviral activity under neutral pH. IONzymes have been well known with peroxidase-like and catalase-like activities, which regulate ROS balance in cellular or bacterial system. However, the two activities were invalid in the antiviral reaction. Wang et al. has reported that Fe_3_O_4_ nanoparticles induced lipid peroxidation by peroxidase-like activity which requires acidic pH and the presence of H_2_O_2_^22^. However, in our work Fe_3_O_4_ nanoparticles could induce lipid peroxidation under neutral pH, indicating that the catalysis is distinct from the peroxidase process. Therefore, we deduce that IONzymes may possess the lipoxidase-like activity in the process of inducing lipid peroxidation to destruct virus.

We found that IONzymes themselves rather than their supernatants destroyed viral particles (Figure S17). In contrast, our recent study reported that Cys-nFeS Nanozymes release polysulfanes into supernatants for antibacterial activity^19^. In addition, we excluded the adsorption effect of IAVs on IONzymes in viral inactivation process. The non-specific adsorption of IAVs on IONzymes may provide false-positive antiviral data because we analyzed virus titers using the samples after IONzymes being removed. To check it, IONzymes were mixed with DyLight 488-labeled IAVs for 2 h and the supernatant and sediments were collected to perform immunofluorescence detection, respectively. We found that fluorescent-labeled viruses were largely existed in supernatants rather than in sediments (Figure S18), suggesting that the decrease of virus titer in supernatants was not due to that IONzymes adsorbed viral particles. Therefore, these analyses confirmed that the viral inactivation was from IONzymes themselves.

Importantly, IONzymes specifically act on viruses containing a lipid envelope, while showing little effect on non-enveloped viruses (PCV-2). In addition, it has previously been shown that polyunsaturated fatty acids (PUFA)-containing phospholipids found in membranes that are highly vulnerable to lipid peroxidation attack^38^. Lipid envelope of influenza virus enriched with PUFA-phospholipids^39^ might be more sensitive to lipid peroxidation of IONzymes. Hemagglutinin and NA are membrane proteins which are responsible for virus infection to host cells and M1 is a matrix protein to form a coat inside the viral envelope^40^-^42^. The lipid peroxidation may directly damage the transmembrane counterpart of hemagglutinin and NA or cause a structure change due to envelope disintegration. The western blot analysis verified that the three proteins were destructed. In contrast, we found that peroxidation cannot impact nucleoprotein (NP), an internal influenza virus protein, encapsulating the virus genome to form a ribonucleoprotein particles (RNP) for the purposes of transcription and packaging^43^. Therefore, our results indicated that the peroxidation damage ignited by IONzymes in viral envelope only passes on the transmembrane proteins and adjacent matrix protein. Previous a study reported that lipid peroxidation led to the disintegration of lipid membrane and the production of lipid-derived radicals, which ultimately damage the proteins^44^, implying that IONzymes induce the lipid peroxidation of viral lipid envelope and may further locally produce an amount of radicals to destroy neighboring proteins.

Importantly, IONzymes inactivate a broad spectrum of influenza A viruses. IAVs can be classified into various subtypes on the basis of antigenic differences in two major surface glycoproteins: hemagglutinin and NA protein, at least 18 hemagglutinin subtypes (H1 to H18) and 11 NA subtypes (N1 to N11) have been detected^45^. The common feature is that all subtypes of IAVs contain a typical lipid envelope. In addition, membrane-proximal cytoplasmic tails of HA and NA are highly conserved in sequence in all subtypes of influenza virus^46^, ^47^. IONzymes treatment remarkably disintegrate envelope and destruct hemagglutinin and NA, we hypothesize that IONzymes are active against other viruses containing a lipid envelope.

It has been well known that facemasks are very important to protect people from influenza infection in crowded spaces. For instance, live-poultry markets (LPMs) allows a high frequency of human-poultry contact and results in a five H7N9 influenza epidemic waves in China 33. Therefore, such protective equipment is needed to intercept virus transmission from poultry to human. However, most facemasks only provide isolation protection for influenza virus but cannot inactivate the adsorbed virus. This may not only provide insufficient protection to the users, but also become a new contaminated source with high spreading risk because virus accumulated on facemasks still remains infectivity^48^. Our IONzyme-loaded facemask possesses an outstanding characteristic to fast inactivate multiple IAVs within the mask, implying potential broad applications of IONzymes in bioprotective PPEs against the emerging influenza threats, especially in high-risk places, such as hospitals and LPMs.

Although IONzymes demonstrated high antiviral activity in our study, the catalytic efficiency should to be further improved to allow its use in practical applications. Currently, high concentration of IONzymes and long incubation times are required in viral inactivation, indicating the activity may not be sufficiently high. Therefore, the activity of IONzymes need to be improved through adjusting nanoparticle size, component or surface modification, or designing a novel single-atom nanozyme^49^ in the future study. In addition, biosafety of IONzymes must be assessed prior use for practical applications.

## Conclusion

In summary, our findings reveal that IONzymes effectively induced lipid peroxidation in viral envelope and dysfunction of neighboring proteins responsible for host infection. IONzymes may perform lipoxidase-like activity in the antiviral process. This viral inactivation via liperoxidation may contribute to understanding antiviral property of iron oxide nanoparticles and other inorganic nanomaterials. In addition, as a typical nanomaterial, IONzymes exhibit features such as multifunctionality, high biocompatibility, high stability and low cost, which will render them competent for serving as a scalable biocidal part of equipment protective against viral infection. Therefore, we propose that IONzymes represent a general antiviral agent against influenza viruses and other enveloped viruses, such as HIV, Ebola virus, Zika virus, West Nile virus, and Nipah virus, all of which represent considerable challenges to national healthcare systems.

## Methods

### Ethics Statement

All of the animal studies were approved by the Jiangsu Administrative Committee for Laboratory Animals (Permission number: SYXKSU-2007-0005) and complied with the guidelines for laboratory animal welfare and ethics of the Jiangsu Administrative Committee for Laboratory Animals. All experiments involving live viruses and animals were performed in negative-pressure isolators with high efficiency particulate air (HEPA) filters in animal biosafety level 3 (ABSL-3) facility in accordance with the institutional biosafety manual.

### Viruses and cells

A/mallard/Huadong/S/2005 (SY, H5N1, clade 2.3.4, GenBank: EU195392.1)^50^, other H5 clades, and different hemagglutinin subtype IAVs (H1N1, H2N2, H3N2, H4N6, H6N2, H7N9, H8N4, H9N2, H10N3, H11N2, and H12N5, list in Table S2) were isolated and identified by our laboratory. All IAVs were propagated in 10-day-old specific-pathogen-free (SPF) embryonic chicken eggs. The SY strain was also prepared in MDCK cells. One part of the SY strain was purified on a discontinuous sucrose density gradient according to our previously described method^51^. Fluorescent labeling of influenza virus was performed using our established method described previously^51^. Briefly, viruses were labeled with the fluorescent probe DyLight 488 NHS Ester (Thermo Fisher Scientific) according to the instructions provided by the manufacturer. Unincorporated dye was removed by using commercial fluorescent dye removal columns (Thermo Fisher Scientific). Labeled viruses were stored at 4 ℃ and used within 2 days. Newcastle disease virus (NDV, envelope virus) 52 and Porcine circovirus 2 (PCV-2, non-enveloped virus) 53 were also isolated, identified, and stored by our laboratory. MDCK cells, and porcine kidney (PK-15) cells were maintained in Dulbecco's modified Eagle's medium (DMEM, Gibco, New York, USA) supplemented with 10% fetal bovine serum (FBS, Foundation, Gemini) at 37 ℃ under 5% CO_2_.

### Synthesis of IONzymes

IONzymes were prepared according to our previously described method^13^. IONzymes were synthesized in one-step in a solvothermal system by combining FeCl_3_ and sodium acetate (NaAc) in ethylene glycol. The size distribution of these IONzymes has been characterized previously^54^. Briefly, 0.82 g of FeCl_3_ was dissolved in 40 mL of ethylene glycol to form a clear solution. Next, 3.6 g of NaAc was added to the solution with vigorous stirring for 30 min. The mixture was then transferred to a 50 mL teflon-lined stainless-steel autoclave and left to react at 200 ℃ for 12 h. After the autoclave cooled to room temperature, the black precipitate was collected, rinsed several times using ethanol and then dried at 60 ℃. The synthesized nanoparticles were characterized using scanning electron microscopy (SEM, S-4800, Hitachi, Japan) and transmission electron microscope (TEM, Tecnai 12, Philips, Netherlands). The enzyme kinetics for peroxidase-like activities of IONzymes were also evaluated.

### Determination of lipid peroxidation

MDA levels, a reliable marker of lipid peroxidation, were measured using thiobarbituric acid (TBA). Different concentrations of IONzymes were mixed with liposomes (3 mg/mL) for 2 h, or IONzymes (4 mg/mL) was mixed with liposomes (4 mg/mL) for different times. After centrifugation, supernatant was collected. In addition, IONzymes were mixed with IAVs (H5N1, SY strain) for 2 h. The magnet was placed under the pipes to pull down IONzymes and then supernatant was also collected to detect the levels of lipid peroxidation using a commercial MDA detection kit according to the manufacturer's instruction (Nanjing Jiancheng Bioengineering Institute, Nanjing, China). MDA concentrations were calculated on the basis of the absorbance of TBA reactive substances (TBARS) at 530 nm.

### Transmission electron microscopy

Samples (supernatant) were primary-fixed with 2.5% glutaraldehyde at 4 °C and then negatively stained with 2% uranyl acetate. The images of influenza virus were captured using a Tecnai 12 transmission electron microscope (Philips, Netherlands). Digital images were acquired using a blazing fast CCD camera system (Gatan, USA).

### Western blot

Samples (supernatant) were lysed with RIPA buffer (Beyotime, Nantong, China) containing protease inhibitor. The lysate was centrifuged at 12,000 g for 10 min at 4 °C and the protein concentrations were determined and calibrated (BCA protein assay kit, Pierce, USA). Protein extracts were resolved on SDS-polyacrylamide gels, transferred to polyvinylidene fluoride membranes, blocked with PBS containing 0.05% Tween (PBST) and 5% skimmed milk powder, and probed with antibodies specific for mAb 3A9 (anti-hemagglutinin of H5N1 IAV)^55^, mouse serum (anti-M1 of H5N1 IAV)^55^, pAb (anti-NA of H5N1 IAV)^56^, and pAb (anti-NP of IAV, Sino Biological Inc, China), followed by incubation with horseradish peroxidase-conjugated goat anti-mouse antibodies (EMD Chemicals). Protein bands were visualized using enhanced chemiluminescence (Thermo Scientific, USA) on radiographic film.

### Hemagglutination assay and TCID_50_ detection

In order to assess the antiviral activity of IONzymes against IAVs, different concentrations of IONzymes were mixed with IAVs for different time. The magnet was placed under the pipes to pull down IONzymes and then supernatant was collected to detect viral titers by hemagglutination (HA) assay and 50% tissue culture infectious doses (TCID_50_) assay. Briefly, for HA assay, 25 μL of PBS was added into all of the experiment's 96 “V”-shaped wells. 25 μL of each supernatant was dispensed into the first well (column 1) and thoroughly mixed, and then the 25 μL mixture in column 1 was transferred to column 2. Two-fold serial dilutions of the samples from column 2 to column 11 were performed. The last 25 μL of fluid was discarded, with column 12 serving as PBS control. Subsequently, 25 μL of PBS was again added into all of wells. 25 μL of 1% chicken red blood cells (cRBCs) suspension was added and mixed to all of the wells. The viral HA titers were evaluated after the plates had been incubated for 10 min at 37 ℃.

To measure the 50% tissue culture infectious doses (TCID_50_), the individual supernatants were serially diluted 10-fold from 10^-1^ to 10^-9^, and each dilution (10^-5^-10^-9^) was inoculated into confluent MDCK cell monolayers by using 96-well plates under 37 °C for 1 h. After 1 h, the monolayer was rinsed with PBS, overlaid with medium (1% FBS in DMEM) and incubated at 37 °C for 72 h. To identify influenza virus positive wells, the HA assay was performed^57^. The Log_10_TCID_50_ per 0.1 mL was calculated using the Reed-Muench method as described previously^27^.

### Neuraminidase activity assay

Supernatants were analyzed using a neuraminidase assay kit (Beyotime Institute of Biotechnology, Nantong, China) according to the manufacturer's instructions. Briefly, 10 μL supernatant were mixed with 70 μL detection buffer, 10 μL NA fluorogenic substrate and 10 μL double-distilled water. Fluorescence was released with an emission wavelength of 460 nm and an excitation wavelength of 360 nm and monitored using a multifunctional microplate reader (Tecan). NA activity is shown as the intensity of fluorescence above the background values for supernatant without virus.

### Immunofluorescence

MDCK cells were infected with IONzyme-treated IAVs at an MOI of 1. At 24 h p.i., the cells were fixed with PBS containing 4% paraformaldehyde for 20 min, saturated with PBS containing 0.2% Triton X-100 for 5 min, and then blocked with 5% bovine serum albumin in PBS for 1 h. Cells were then washed three times with PBS and incubated at 37 °C for 1 h with mAb 3A9 (anti-hemagglutinin of H5N1 IAV), followed by incubation with fluorescein isothiocyanate (FITC) -conjugated goat anti-mouse IgG (Sigma, MO, USA). After being washed, the cells were observed using a Leica fluorescence microscopy.

### Apoptosis assay

MDCK cells were infected with IONzyme-treated or untreated IAVs (SY strain) at an MOI of 1. At 24 h p.i., both infected and noninfected cells were treated with trypsin and were slightly washed three times with PBS. According to the manufacturer's instructions (BD Biosciences, San Jose, CA, USA), an aliquot of cells (~10^6^) was stained with FITC-conjugated annexin V and propidium iodide (PI) for 15 min at room temperature in the dark. The stained cells were then analyzed by a caliber flow cytometry (Becton Dickinson, Franklin Lakes, NJ, USA). Annexin V possesses high affinity for phosphatidylserine (PS), which is translocated from the inner to the outer leaflet of the plasma membrane in the process of apoptosis. PI cannot permeate live cells and early apoptotic cells but stains necrotic cells, binding tightly to the nucleic acids in the cell. As a result, early apoptotic cells are characterized by annexin V^+^ and PI^-^ staining, later apoptotic cells by annexin V^+^ and PI^+^ staining, whereas necrotic cells by annexin V^-^ and PI^+^. The cell proportions were analyzed using FlowJo V10 software (FlowJo LLC, Ashland, OR, USA).

For immunofluorescence detection of PCV-2, PK-15 cells were infected with IONzyme-treated or untreated PCV-2 at MOI=0.5 for 24 h. After washing, fixation, saturation, and blocking, the cells were incubated at 37 °C for 1 h with using a pig polyclonal antiserum (VMRD, Washington, USA)^58^, followed by incubation with FITC-conjugated rabbit anti-pig antibody (Southern Biotech, Birmingham, USA). The cells were washed and examined under a fluorescence microscope. Cells positive for PCV-2 viral antigens were counted in six fields of view.

### Pathogenicity of IONzyme-treated IAVs in mice

Four groups of 6-week-old female BALB/c mice were lightly anesthetized and then inoculated intranasally with10^6^ EID_50_ of each IONzyme-treated or untreated H5N1 IAVs (SY strain) in 50 μL of PBS. Animals were monitored daily for weight loss and mortality over a period of 14 days as described previously^59^. Three mice of each group were euthanized at 7 days p.i., and lung tissues were fixed for histopathological examination^60^, or aseptically for virus titration. Each tissue sample was homogenized in 1 mL of PBS containing antibiotics and centrifuged at 6,800 rpm for 10 min, and 0.1 mL of supernatant was used to inoculate SPF chicken embryo from initial dilutions of 1:10. Virus titers were calculated as described^27^.

### Application of IONzymes in anti-IAV facemasks

Basing on the traditional facemasks with absorbent filter cotton, different concentrations of IONzymes (0.8 mg/cm^2^, 0.4 mg/cm^2^, 0.2 mg/cm^2^, and 0.1 mg/cm^2^) were added on the third layer of selected area (5 cm^2^), respectively. After drying by airflow for 30 min, IAVs was sprayed onto the eighth layer (the outermost layer). After incubating for 0.5 or 1 h, both the control area (equal volume PBS) and the IONzyme area were placed into a tube containing 200 μL PBS. After washing and extrusion, viral suspensions were harvested, and the population of viable virus was then measured using HA and TCID_50_ methods.

### Statistical analysis

Results were expressed as the means ± SD and analyzed with GraphPad Prism 8 software (San Diego, CA). Unpaired Student's two-sided *t*-test was employed to determine the differences between the two groups. *P< 0.05, **P< 0.01. Data were combined from at least three independent experiments unless otherwise stated.

## Supplementary Material

Supplementary figures and tables.

## Abbreviations

IAV: Influenza A virus
IONzymes: iron oxide nanozymes
NA: neuraminidase
M1: matrix protein 1
HA: hemagglutination
TCID_50_: 50% tissue culture infectious doses
MDA: maleic dialdehyde
TMB: 3,3',5,5'-tetramethyl benzidine
H_2_O_2_: hydrogen peroxide
SEM: scanning electron microscopy
TEM: transmission electron microscopy
NP: nucleoprotein
BSA: bovine serum albumin
HRP: horseradish peroxidase
NDV: Newcastle disease virus
PCV-2: Porcine circovirus type 2
p.i.: post infection
MOI: multiplicity of infection
MFI: mean fluorescence intensity
PI: propidium iodide
PBS: phosphate-buffered saline
H&E: hematoxylin and eosin
SPF: specific pathogen free
EID_50_: 50% embryo infectious doses
LPMs: live-poultry markets
PPEs: personal protective equipments
ROS: reactive oxygen species
HEPA: high efficiency particulate air
ABSL-3: animal biosafety level 3
MDCK: Madin-Darby canine kidney
PK-15: porcine kidney 15
DMEM: Dulbecco's modified Eagle's medium
FBS: fetal bovine serum
NaAc: sodium acetate
TBA: thiobarbituric acid
TBARS: TBA reactive substances
cRBCs: chicken red blood cells
PS: phosphatidylserine
FITC: fluorescein isothiocyanate
