# Supplementary material for: Catalytic inactivation of influenza virus by iron oxide nanozyme
Source: Theranostics. 2019 Sep 21;9(23):6920–35. doi: 10.7150/thno.35826 (PMC6815955; doi:10.7150/thno.35826)
Supplement: Supplementary file 1 — Supplementary figures and tables. [file thnov09p6920s1.pdf]

Supporting Information

Catalytic inactivation of influenza virus by iron oxide nanozyme

Tao Qin[1,3,5†], Ruonan Ma[1†], Yinyan Yin[2,3], Xinyu Miao[1], Sujuan Chen[1,3,5], Kelong Fan[4], Juqun Xi[2], Qi Liu[2], Yunhao Gu[2], Yuncong Yin[1], Jiao Hu[1,3,5], Xiufan Liu[1,3,5], Daxin Peng[1,3,5,6]*, and Lizeng Gao[2,3,4]*

[1] College of Veterinary Medicine, Yangzhou University, Yangzhou, Jiangsu, 225009, PR China.

[2] Institute of Translational Medicine, School of Medicine, Yangzhou University, Yangzhou, Jiangsu, 225001, PR China.

[3] Jiangsu Co-Innovation Center for the Prevention and Control of Important Animal Infectious Disease and Zoonoses, Yangzhou, Jiangsu, 225009, PR China.

[4] CAS Engineering Laboratory for Nanozyme, Institute of Biophysics, Chinese Academy of Sciences, Beijing, 100101, China.

[5] Jiangsu Research Centre of Engineering and Technology for Prevention and Control of Poultry Disease, Yangzhou, Jiangsu, 225009, PR China.

[6]Joint Laboratory Safety of International Cooperation of Agriculture&Agricultural-Products, Yangzhou, Jiangsu 225009, PR China.

[†]These authors contributed equally to this work

[*]Corresponding authors: pengdx@yzu.edu.cn (Daxin Peng) and lzgao@yzu.edu.cn (Lizeng Gao)

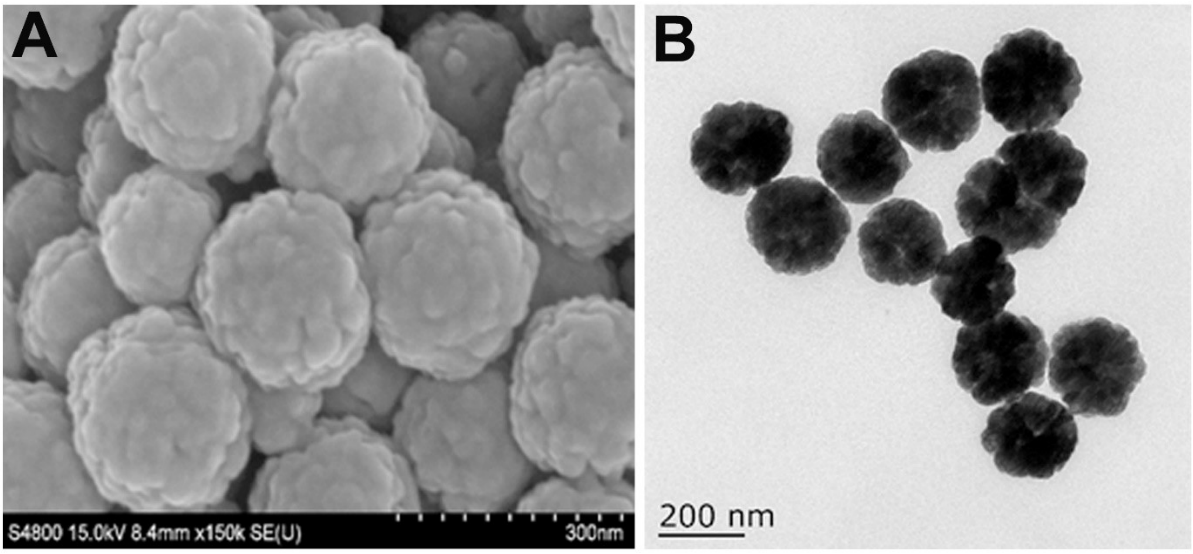

Figure S1. **Characterizations of IONzymes (A)** The SEM image of the prepared IONzymes. Scale bar: 300 nm. **(B)** The TEM micrograph of the prepared IONzymes, Scale bar: 200 nm. Representative images are shown.

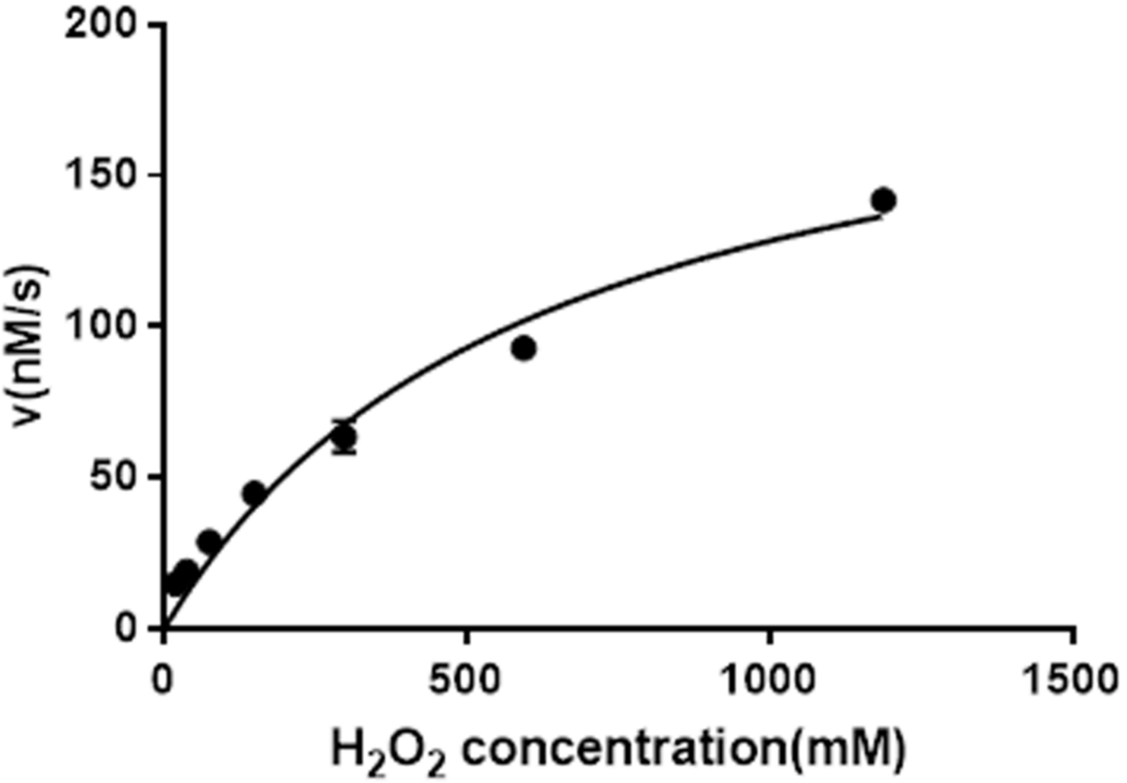

Figure S2. **Michaelis-Menten kinetics for peroxidase-like activity of IONzymes towards H_2O_2**. The reaction was conducted in NaAc buffer (0.1 M, pH 4.5) containing 10 μg/mL IONzymes and 833.33 μM TMB with variable H_2O_2 concentrations. Data shown represent the means±s.d. of three independent experiments.

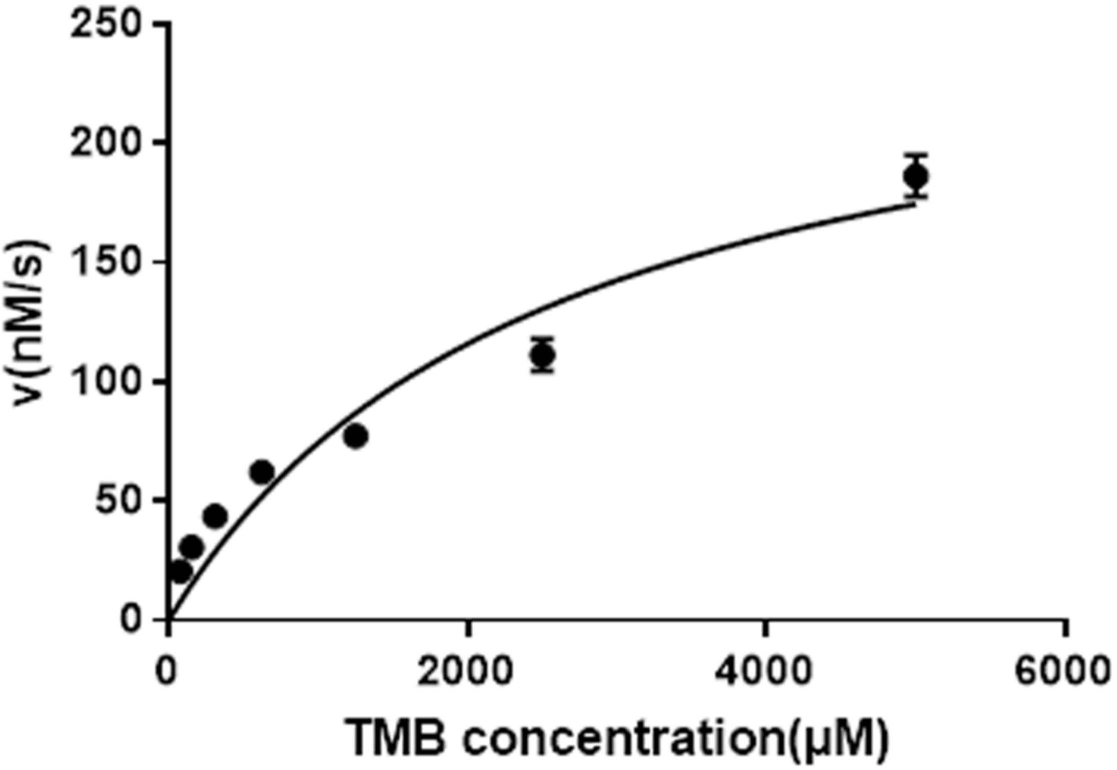

Figure S3. **Michaelis-Menten kinetics for peroxidase-like activity of IONzymes towards TMB.** The reaction was conducted in NaAc buffer (0.1 M, pH 4.5) containing 10 μg/mL IONzymes and 148.46 mM H_2O_2 with variable TMB concentrations. Data shown represent the means±s.d. of three independent experiments.

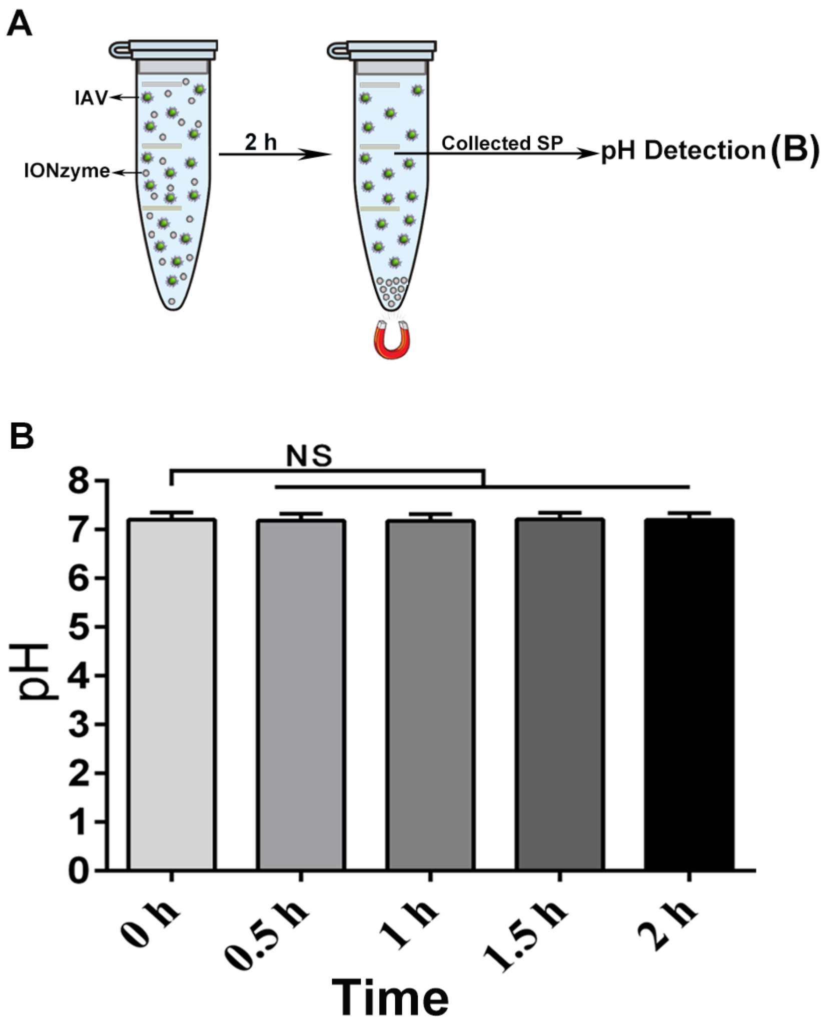

Figure S4. **pH detection during the interaction between IONzymes and IAVs. (A-B)** IONzymes (4 mg/mL) were mixed with IAVs (H5N1 SY strain) for different time. The magnet was placed under the pipes to pull down IONzymes and then supernatant was collected to detect pH value by pH Meter. Data shown represent the means±s.d. of three independent experiments. Statistical significance is assessed by unpaired Student's two-sided *t*-test to the control group (0 h). *NS* represents no significant difference.

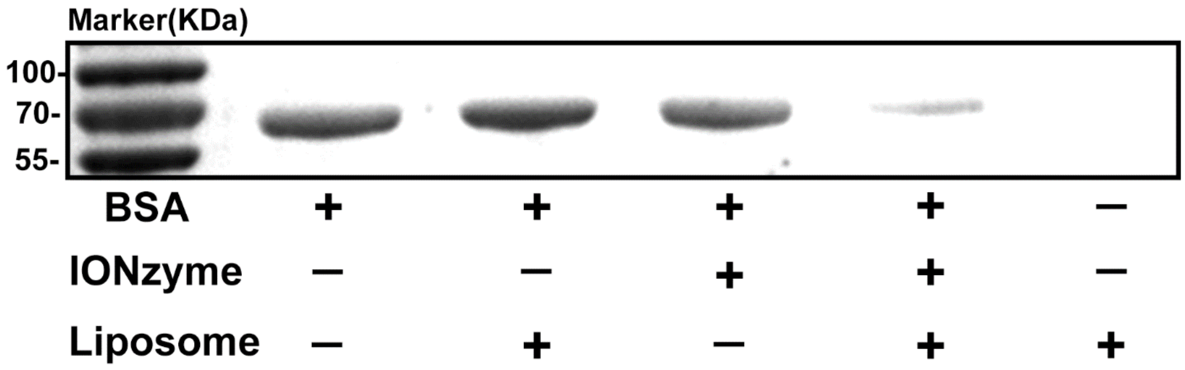

Figure S5. **The destruction of bovine serum albumin (BSA) in presence of liposome after IONzymes incubation.** Firstly, bovine serum albumin (BSA) (1.08 mg) was sufficiently mixed with 1mL liposomes (3.5 mg/mL). Subsequently, IONzymes (4 mg/mL) were added and mixed with above 0.2 mL mixture at 37 °C under neutral pH. After 2 h, the magnet was placed under the pipes to pull down IONzymes and then supernatant was collect to perform SDS-PAGE and Coomassie staining. All experiments were repeated in triplicate with a representative image shown.

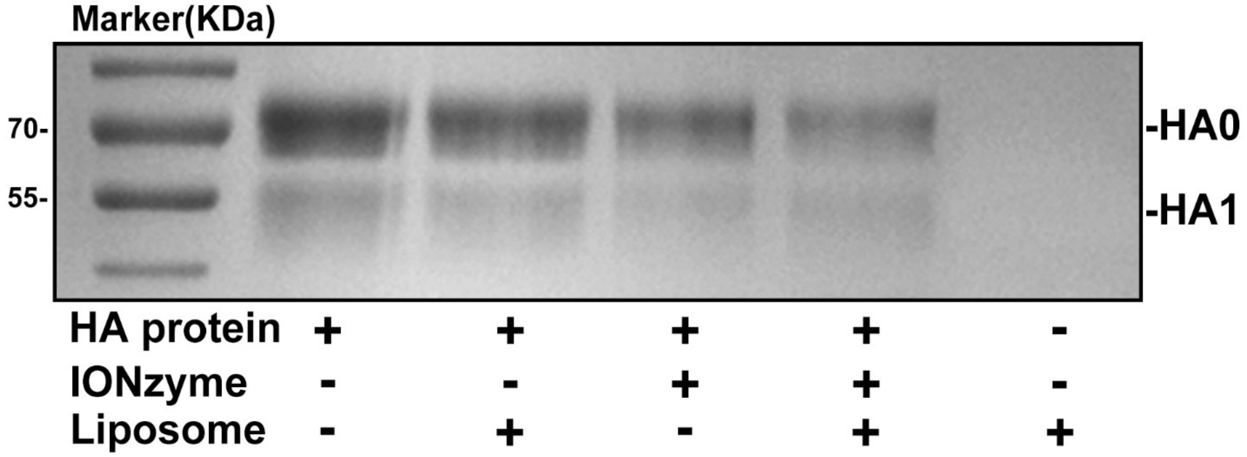

Figure S6. **The destruction of hemagglutinin protein in presence of liposomes after IONzymes incubation.** The destruction of hemagglutinin protein (A/AnHui/1/2005 (H5N1)) in presence of liposomes after IONzymes incubation. Firstly, hemagglutinin protein (0.52 mg) was sufficiently mixed with 1 mL liposomes (3.5 mg/mL). Subsequently, IONzymes (2 mg/mL) were added and mixed with above 0.2 mL mixture at 37 °C under neutral pH. After 2 h, the magnet was placed under the pipes to pull down IONzymes and then supernatant was collect to perform western blot analysis. All experiments were repeated in triplicate with a representative image shown.

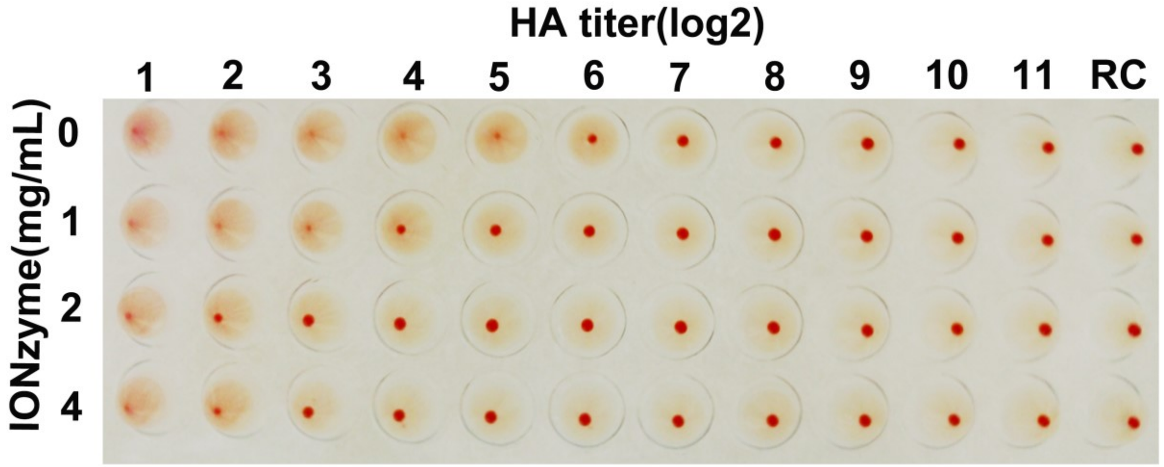

Figure S7. **Evaluation of hemagglutination of IAVs.** Different concentrations of IONzymes were mixed with chicken embryos-produced IAVs (H5N1 SY strain) for 2 h. The magnet was placed under the pipes to pull down IONzymes and then supernatant was collected to detect hemagglutination (HA) titers. RC represents the control of red cells. The picture is presented as one of three independent experiments.

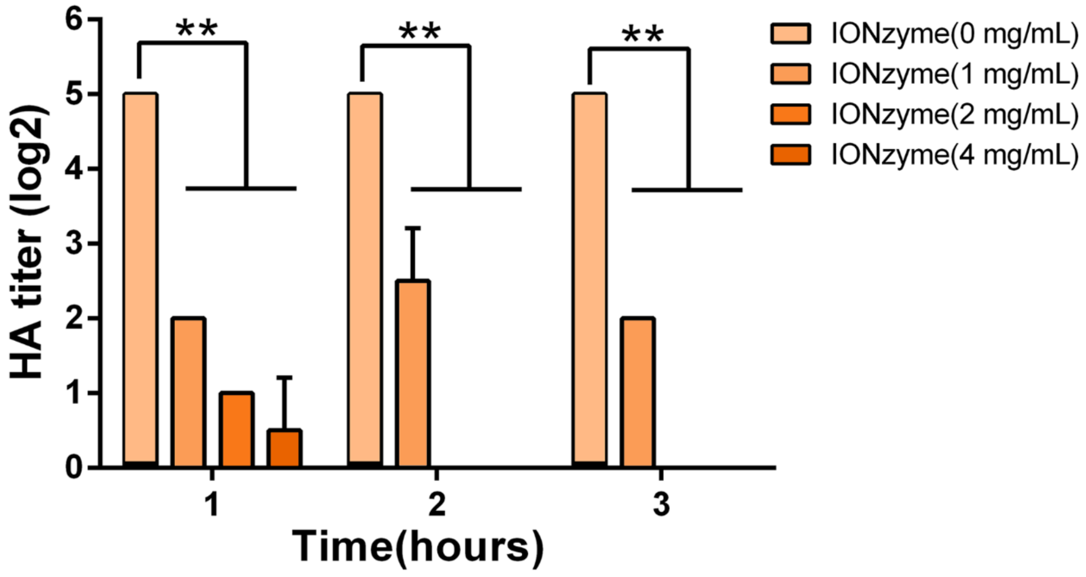

Figure S8. **Evaluation of HA titer of cell-produced IAVs.** Different concentrations of IONzymes were mixed with cell-produced IAVs (H5N1 SY strain) for different time. The magnet was placed under the pipes to pull down IONzymes and then supernatant was collected to detect HA titer. Data shown represent the means±s.d. of three independent experiments. Statistical significance is assessed by unpaired Student's two-sided *t*-test to the control group (IONzymes, 0 mg/mL). $^{**}P<0.01$.

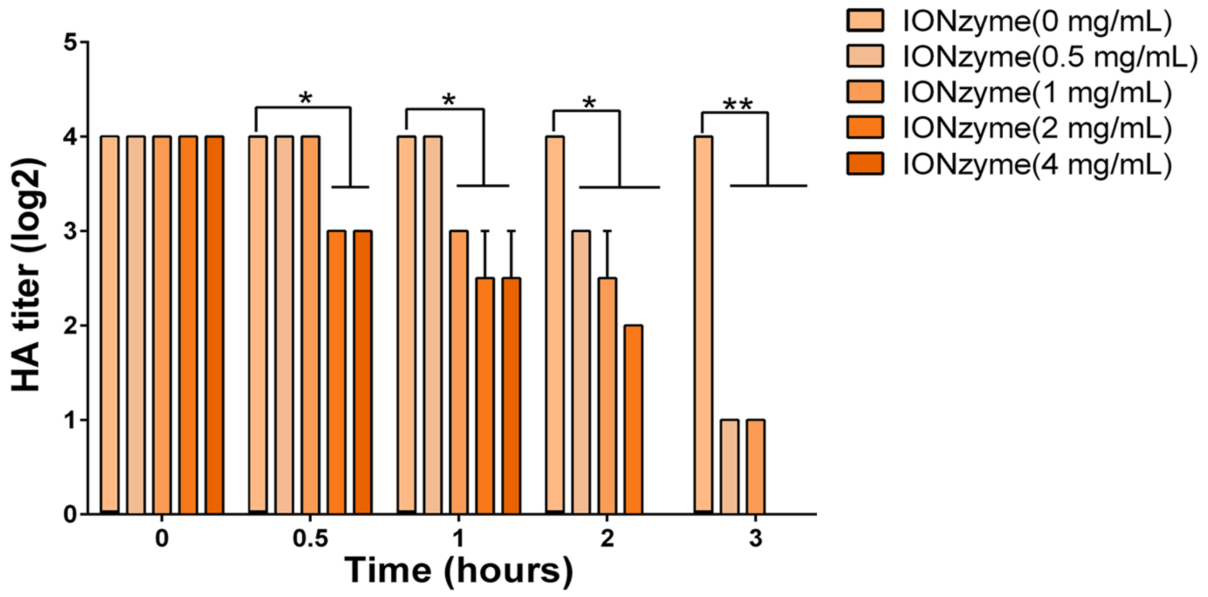

Figure S9. **Evaluation of HA titer of purified IAVs.** Different concentrations of IONzymes were mixed with purified IAVs (H5N1 SY strain) for different time. The magnet was placed under the pipes to pull down IONzymes and then supernatant was collected to detect HA titer. Data shown represent the means±s.d. of three independent experiments. Statistical significance is assessed by unpaired Student's two-sided *t*-test to the control group (IONzymes, 0 mg/mL). **P<0.05; **P<0.01.*

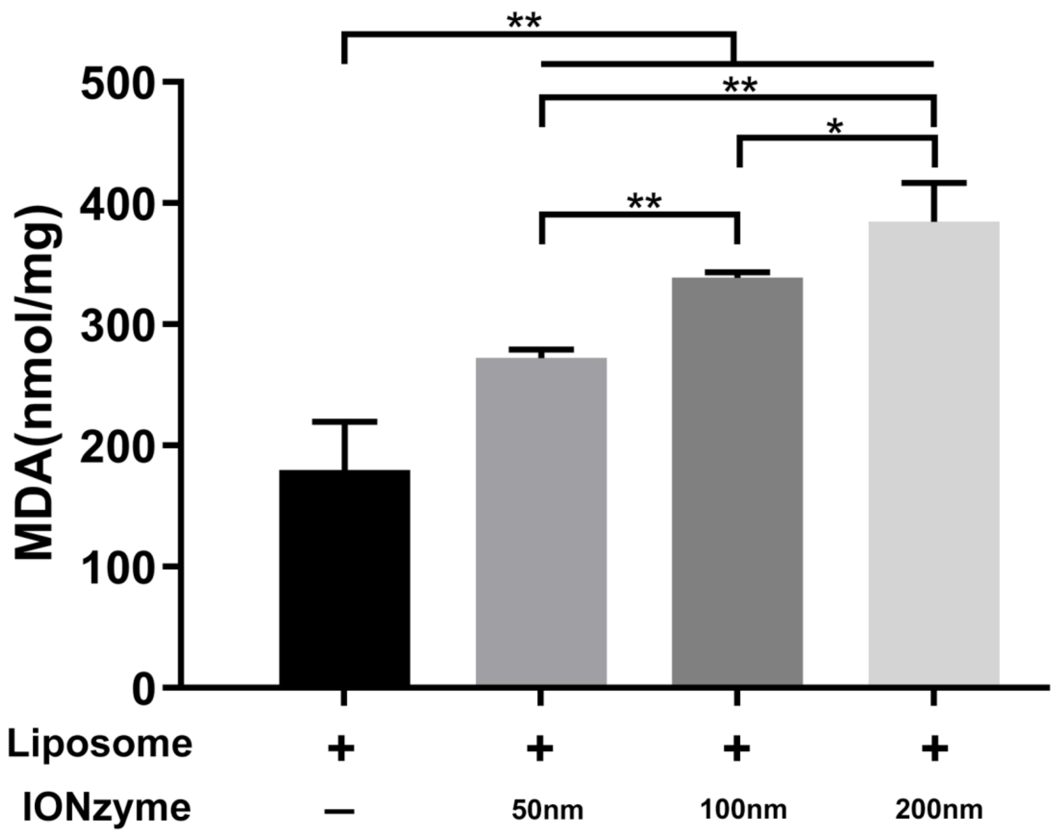

Figure S10. **The level of lipid peroxidation of IONzymes with different size.** Liposome (4 mg/mL) was treated by IONzymes (3 mg/mL) with different scales (50 nm, 100 nm, and 200 nm) for 2 h. The level of lipid peroxidation (MDA detection) was detected by commercial MDA detection kit according to the manufacturer's instruction. Data shown represent the means±s.d. of three independent experiments. Statistical significance is assessed by unpaired Student's two-sided *t*-test. **P*<0.05, ***P*<0.01.

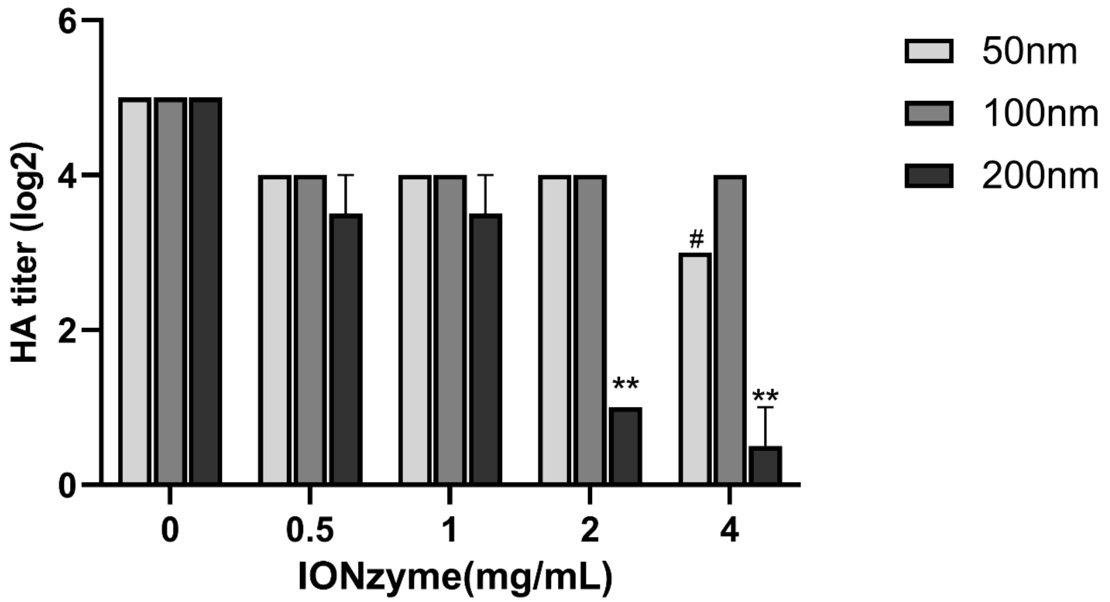

***Figure S11.* Anti-IAV activity of IONzymes with different size.** Different concentrations of IONzymes (50 nm, 100 nm, and 200 nm) were mixed with IAVs (H5N1 SY strain) for 2 h. The magnet was placed under the pipes to pull down IONzymes and then supernatant was collected to detect HA titer. Data shown represent the means±s.d. of three independent experiments. Statistical significance is assessed by unpaired Student's two-sided *t*-test to the control group (IONzymes, 0 mg/mL). $^{\#}P<0.05$. $^{**}P<0.01$.

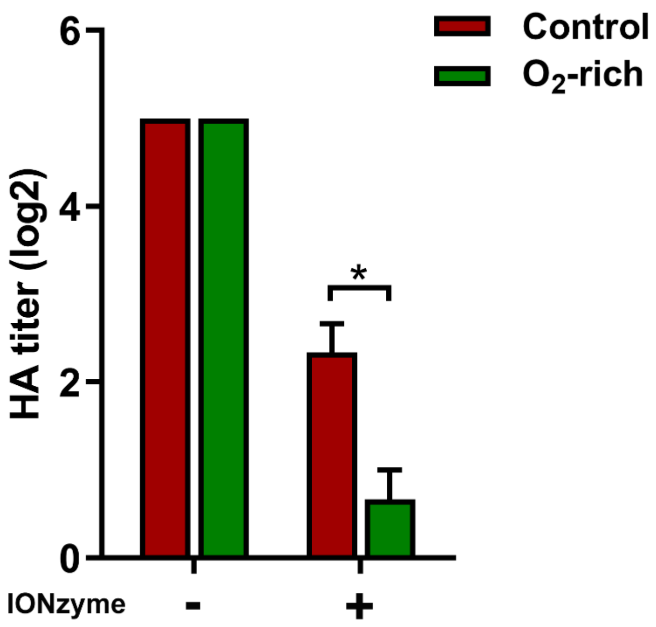

Figure S12. **Anti-IAV activity of IONzymes under O_2-rich conditions.** HA titer of IONzymes (0.5 mg/mL)-treated IAVs under normal or O_2-rich conditions for 3 h. Data shown represent the means±s.d. of three independent experiments. Statistical significance is assessed by unpaired Student's two-sided *t*-test. **P*<0.05.

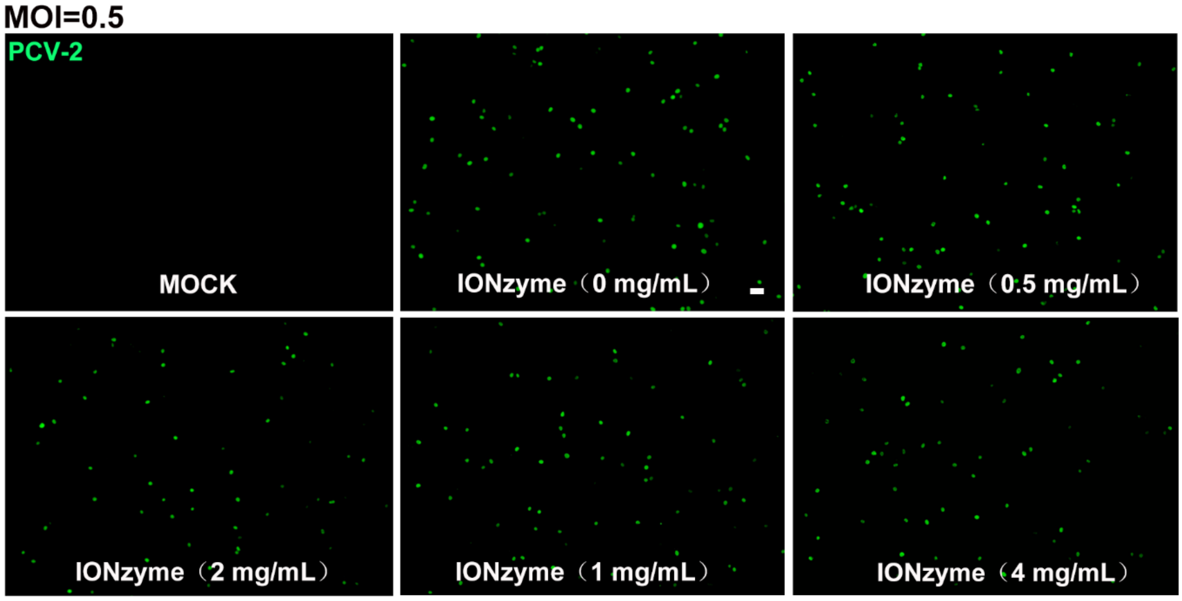

Figure S13. **Antiviral activity of IONzymes against non-enveloped virus.** Different concentrations of IONzymes was mixed with PCV-2 (non-enveloped virus) for different time. The magnet was placed under the pipes to pull down IONzymes and then supernatant was collected to detect the number of PCV2-infected PK15 cells (green) by immunofluorescence. Images are from a representative experiment out of three.

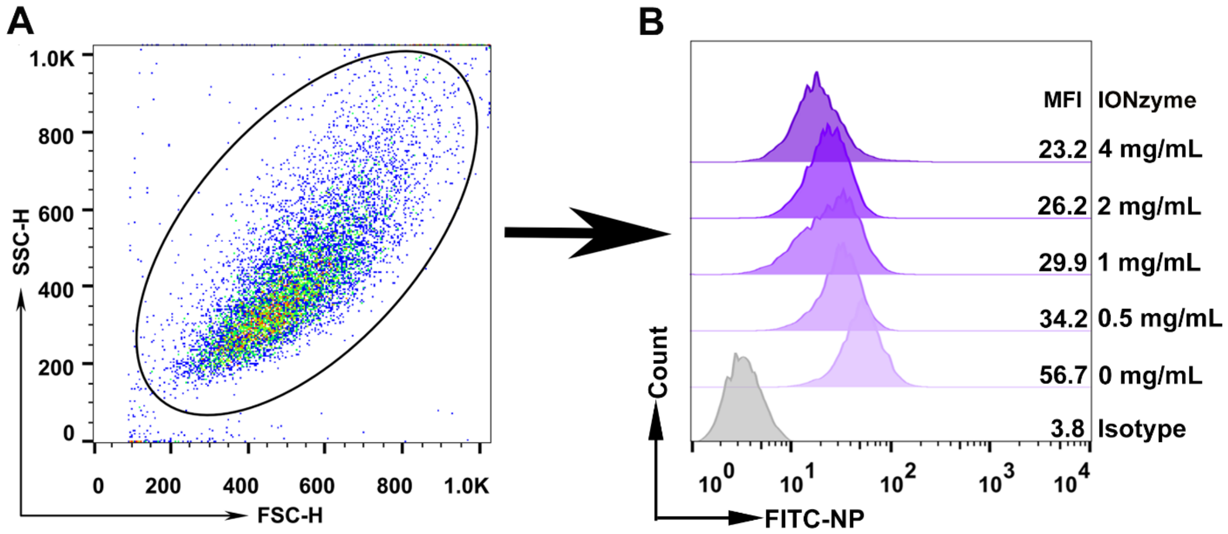

Figure S14. **Attachment ability of IONzyme-treated IAVs *in vitro*.** MDCK cells were attached with IONzyme-treated or untreated H5N1 SY strain (pretreated for 2 h) for 1 h at 4 °C (MOI = 100). The cells were washed, fixed, permeabilized, and labeled FITC-NP antibody (Abcam, ab20921) to detect the expression of NP protein by flow cytometry. MFI, mean fluorescence intensity. **(B)** was gated from **(A)**. The picture is presented as one of three independent experiments.

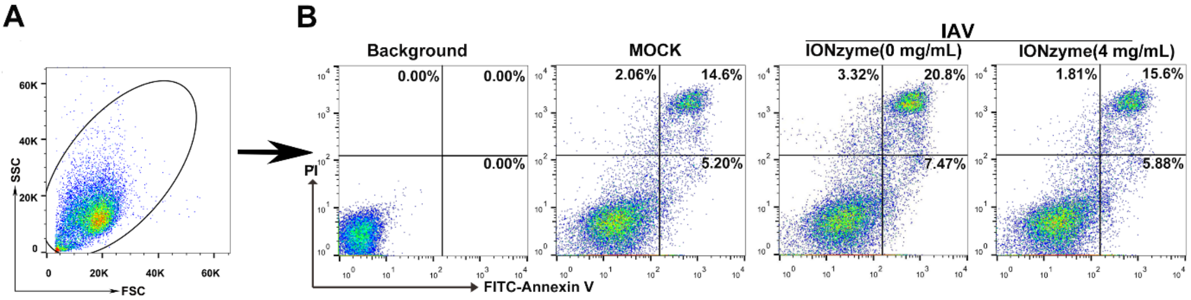

Figure S15. **Evaluation of apoptosis induced by IONzyme-treated IAVs *in vitro*.** MDCK cells were infected with IONzyme-treated or untreated H5N1 IAVs (SY strain) at an MOI of 1 at 24 h p.i.. Cells were stained with FITC-conjugated annexin V and propidium iodide (PI) and then detected by flow cytometry. **(B)** was gated from **(A)**. The picture is presented as one of three independent experiments.

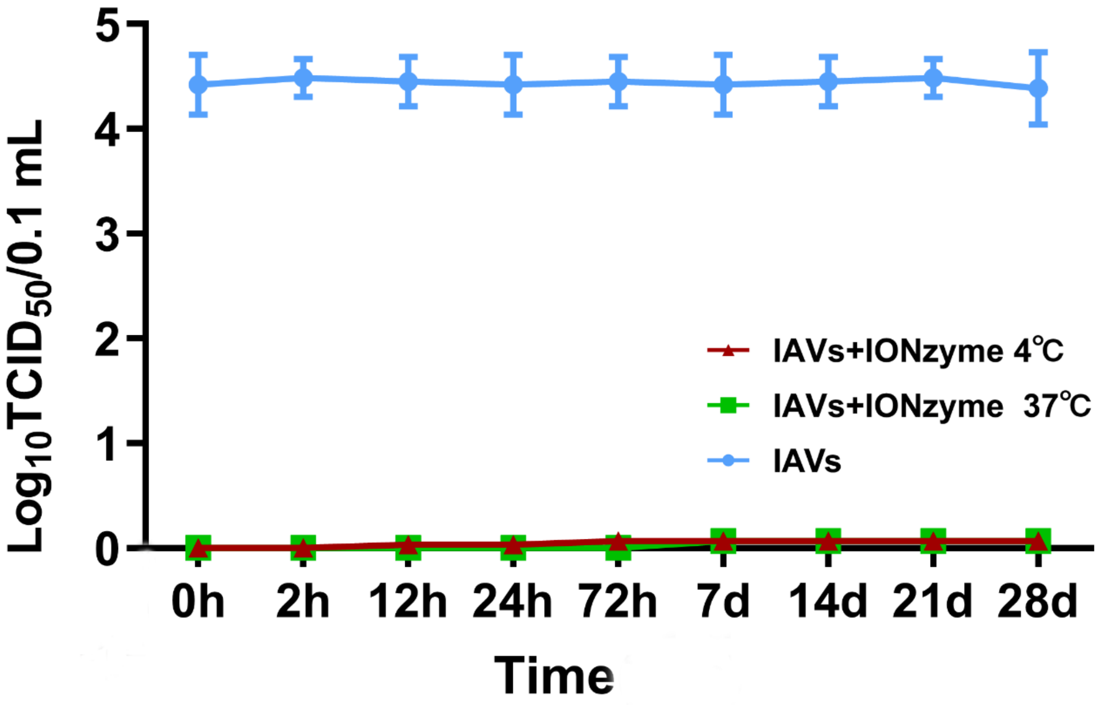

Figure S16. **Antiviral ability of IONzyme-loaded facemask with a long-term storage.** IONzymes (0.8 mg/cm^2) were added on the third layer of selected area (5 cm^2), and then facemasks were stored at 4 °C or 37 °C. At different time points, IAVs (H5N1, SY strain) was sprayed onto the outermost layer of facemask. After standing for 1 h, the population of the living virus on the control area or IONzyme-added area were harvested, and then $TCID_{50}$ titer of IAVs was measured. The data are presented as the mean ± s.d. of three independent experiments.

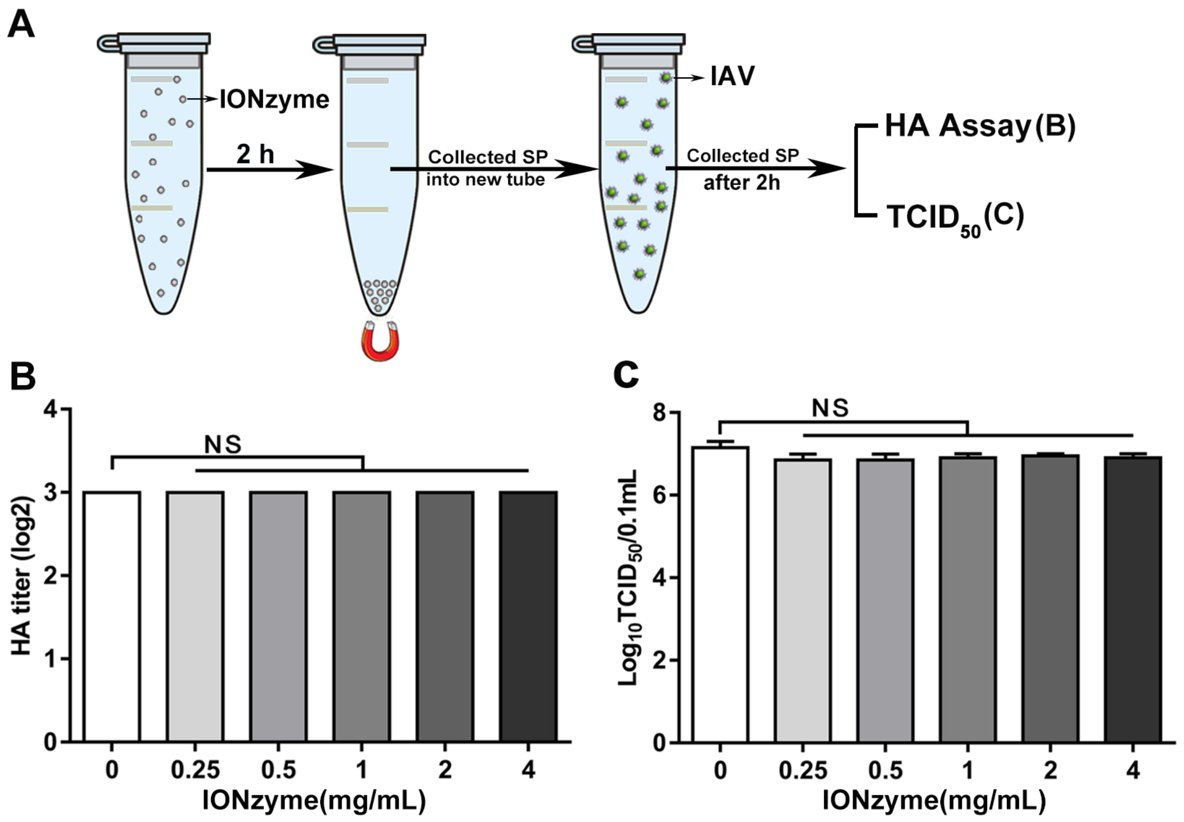

***Figure S17*. Anti-IAV effect of supernatant from IONzymes. (A)** Different concentrations of IONzymes were firstly standed for 2 h, then IONzymes were pulled down by a magnet placed under the pipes, and then supernatants were collected and mixed with IAVs (SY strain) for 2 h, subsequently, HA **(B)** and $TCID_{50}$ **(C)** titers were detected. Data shown represent the means±s.d. of three independent experiments. Statistical significance is assessed by unpaired Student's two-sided *t*-test to the control group (IONzymes, 0 mg/mL). *NS* represents no significant difference.

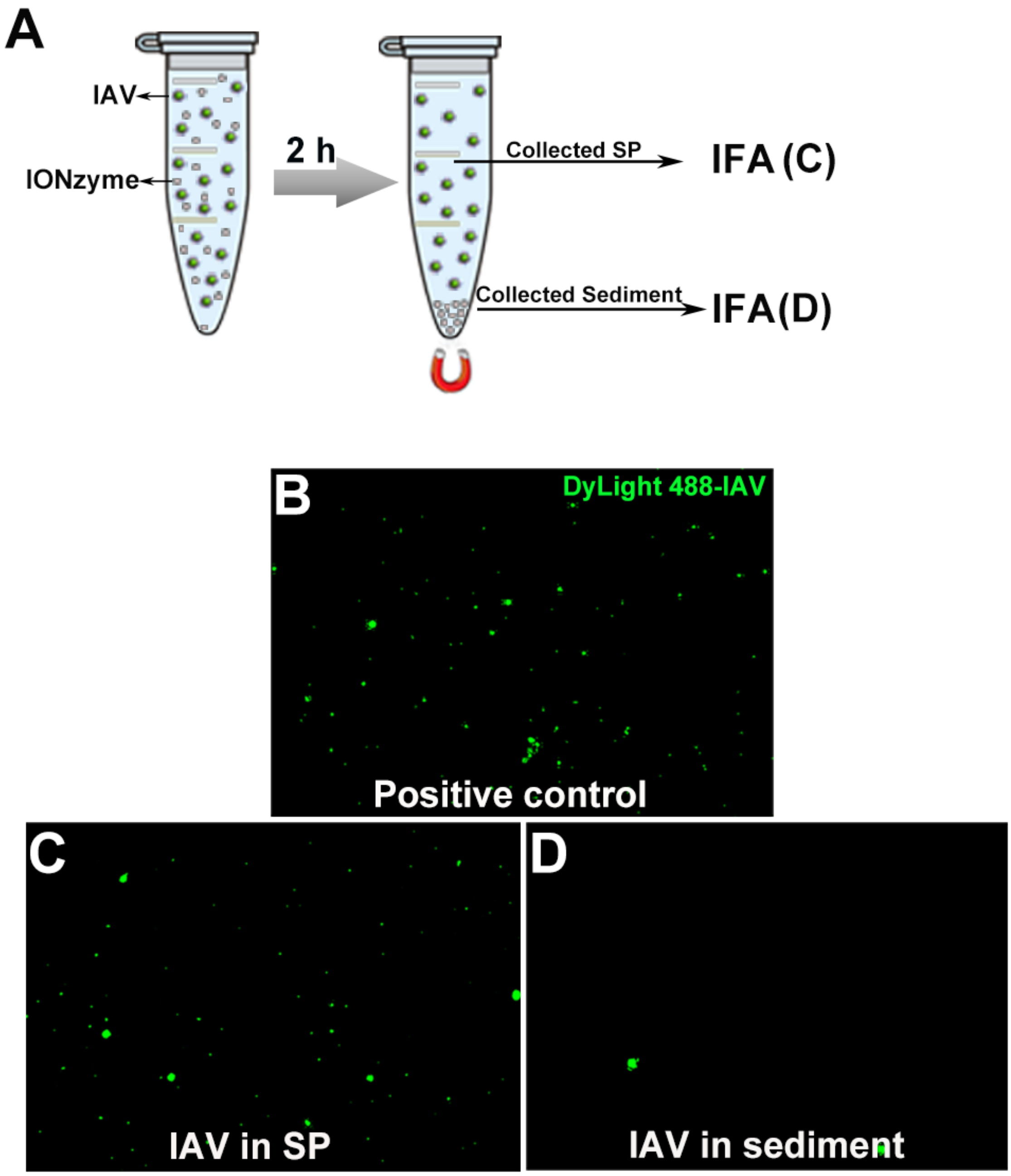

Figure S18. **Virus detection in supernatant and sediment from IONzyme-treated IAVs. (A)** The scheme depicts that IONzymes (4 mg/mL) were mixed with DyLight 488-labeled IAVs (H5N1 SY strain) for 2 h. The magnet was placed under the pipes to pull down IONzymes and then supernatant (SP) **(C)** or sediment **(D)** was collected to observe DyLight 488-IAVs by immunofluorescence. **(B)** Positive control. Green represents IAVs. Images are from a representative experiment out of three.

***Table S1.* Kinetic parameters of IONzymes when H_2O_2 or TMB concentration was variable**

	H_2O_2	TMB
Vmax (nM/s)	207	263.5
Km (mM)	612.2	2.545
Vmax/Km (10^{-6}/s)	0.338125	0.1035

***Table S2.* The strains of the influenza A virus used in this study**

Viruses	Subtype/Clade
A/Swine/Jiangsu/48/2010	H1N1
A/duck/Wuxi/2/2013	H2N2
A/Duck/Eastern China/866/2003	H3N2
A/Duck/Eastern China/160/2002	H4N6
A/Duck/Eastern China/164/2002	H6N2
A/Chicken/Eastern China/JD/2017	H7N9
A/Duck/Eastern China/01/2005	H8N4
A/Duck/Eastern China/01/2000	H9N2
A/Duck/Eastern China/488/2003	H10N3
A/Duck/Eastern China/05/2005	H11N2
A/Duck/Eastern China/37/2015	H12N5
A/Mallard/Huadong/S/2005	H5N1(clade 2.3.4)
A/Duck/Henan/ZK5/2013	H5N2(clade 0)
A/Duck/Shanghai/F1/2012	H5N8(clade 2.3.4)
A/Chicken/Xuyi/XYC5729/2015	H5N1(clade 2.3.2.1)
A/Chicken/Jiangsu/XZXY/2013	H5N2(clade 7.2)
A/Chicken/Guizhou/4/2013	H5N1(clade 2.3.4.4)
